# Jaguar Range‐Wide Connectivity: Prioritising Core Areas and Corridors Between and Within Protected Areas and Indigenous Lands

**DOI:** 10.1111/gcb.71093

**Published:** 2026-09-04

**Authors:** Guilherme Costa Alvarenga, Caroline C. Sartor, Ana Carolina Srbek‐Araujo, Ana Cristina Mendes‐Oliveira, Bart Harmsen, Carlos De Angelo, Carolina Franco Esteves, Claudia Bueno de Campos, Daiana Jeronimo Polli, Diego F. Passos Viana, Diogo Maia Gräbin, Emiliano Donadio, Emiliano E. Ramalho, Esteban Payán, Fernando C. C. Azevedo, Francisco Palomares, George V. N. Powell, Gerardo Ceballos, Grasiela Porfirio, Heliot Zarza, Ivonne Cassaigne, Juliano A. Bogoni, Leonardo Sena, Louise Maranhão, Marcos Roberto Monteiro de Brito, Mathias W. Tobler, Øystein Wiig, Rafael Antelo, Rebecca J. Foster, Ricardo Sampaio, Robin Naidoo, Rodrigo Nuñez, Ronaldo G. Morato, Sandra Petrone Mendoza, Valeria Boron, Wener Hugo Arruda Moreno, David W. Macdonald, Żaneta Kaszta, Samuel A. Cushman

**Affiliations:** ^1^ Wildlife Conservation Research Unit (WildCRU), Department of Biology University of Oxford Oxford UK; ^2^ Grupo de Pesquisa em Ecologia e Conservação de Felinos na Amazônia Mamirauá Institute for Sustainable Development (MISD) Tefé Amazonas Brazil; ^3^ Programa de Pós‐Graduação em Ciência Animal e Programa de Pós‐Graduação em Ecologia de Ecossistemas Universidade Vila Velha Vila Velha Espírito Santo Brazil; ^4^ Laboratório de Ecologia e Zoologia de Vertebrados (LABEV/ICB) Universidade Federal do Pará Belém Pará Brazil; ^5^ Panthera New York New York USA; ^6^ Instituto de Ciencias de la Tierra, Biodiversidad y Ambiente (IBCIA) Universidad Nacional de Río Cuarto and National Scientific and Technical Research Council (CONICET) Río Cuarto Argentina; ^7^ Institute for the Conservation of Neotropical Carnivores Atibaia São Paulo Brazil; ^8^ Programa de Pós‐Graduação em Ecologia e Conservação, Instituto de Biociências—Inbio Universidade Federal de Mato Grosso do Sul Campo Grande Mato Grosso do Sul Brazil; ^9^ Fundación Rewilding Argentina Buenos Aires Argentina; ^10^ WCS Big Cat Program New York New York USA; ^11^ Departamento de Ciências Naturais Universidade Federal de São João del Rei São João del Rei Minas Gerais Brazil; ^12^ Department of Conservation Biology, Doñana Biological Station CSIC Sevilla Spain; ^13^ Wildlife Protection Solutions Denver Colorado USA; ^14^ Laboratorio de Ecología y Conservación de Fauna Silvestre, Instituto de Ecología Universidad Nacional Autónoma de México, Ciudad Universitaria Ciudad de México México; ^15^ Instituto Homem Pantaneiro Corumbá Mato Grosso do Sul Brazil; ^16^ Departamento de Ciencias Ambientales Universidad Autónoma Metropolitana, Unidad Lerma, CBS Lerma de Villada Mexico; ^17^ Primero Conservation Pinetop Arizona USA; ^18^ Centro de Pesquisa de Limnologia, Biodiversidade e Etnobiologia do Pantanal, Laboratório de Mastozoologia, Programa de Pós‐graduação em Ciências Ambientais Universidade do Estado de Mato Grosso—UNEMAT Cáceres Mato Grosso Brazil; ^19^ Mamirauá Institute for Sustainable Development (MISD) Tefé Amazonas Brazil; ^20^ Associação Onçafari São Paulo São Paulo Brazil; ^21^ San Diego Zoo Wildlife Alliance, Conservation Science and Wildlife Health Escondido California USA; ^22^ Natural History Museum University of Oslo Oslo Norway; ^23^ WWF‐Bolivia Santa Cruz Bolivia; ^24^ Centro Nacional de Pesquisa e Conservação de Mamíferos Carnívoros (CENAP‐ICMBio) Atibaia São Paulo Brazil; ^25^ WWF Washington DC USA; ^26^ Institute for Resources, Environment and Sustainability University of British Columbia Vancouver British Columbia Canada; ^27^ Lab. Mastozoología & Alianza Jaguar AC, Facultad de Biología Universidad Michoacana Morelia Michoacán Mexico; ^28^ World Wildlife Fund Inc. (WWF Mexico Programme Office) Ciudad de Mexico Mexico; ^29^ World Wildlife Fund for Nature (WWF) UK, The Living Planet Centre Woking UK; ^30^ Department of Biological Sciences Northern Arizona University Flagstaff Arizona USA

**Keywords:** cumulative resistant kernels, dispersal corridors, landscape connectivity, least‐cost path analysis, *Panthera onca*, resistance surface

## Abstract

Anthropogenic habitat transformation has led to population declines and increasing isolation of populations of many species, particularly top predators, where small and fragmented populations often experience limited dispersal and reduced genetic diversity. Jaguars ( *Panthera onca* ), for example, have lost half of their historical range and most populations outside the Amazon biome are small, isolated and highly threatened. Here, we assess jaguar population connectivity across the species' range by identifying and ranking core areas and dispersal corridors, including linkages among Protected Areas and Indigenous Lands (hereafter, PAs). We used cumulative resistant kernels and factorial Least‐Cost Paths (fLCP) in two complementary approaches: core area‐based and PA‐based connectivity. We identified 307 core areas for jaguar movement, covering ~7.75 million km^2^ (55% of the current range), of which approximately half lies within PAs. The largest and highest‐ranked core area spans ~6.5 million km^2^ across four biomes (Amazon, Llanos, Chaco and Pantanal). We identified 176 dispersal corridors connecting core areas, although connectivity was not continuous across the entire range. Northern populations (Mexico and Central America) showed several connectivity gaps, whereas South America displayed a large, highly interconnected network linking the Amazon, Llanos, Chaco, Pantanal, Cerrado, Caatinga, Iberá and the Atlantic Forest's Green Corridor. In contrast, coastal Atlantic Forest patches appeared largely isolated. The PA‐based analysis predicted 507 potential corridors connecting 905 PAs, including potential links absent from the core area model, particularly across Central America and between the Darién Gap, Amazon and coastal Atlantic Forest. The core‐area approach provides a more biologically realistic representation of current connectivity, while the PA‐based approach complements it by identifying pragmatic opportunities for long‐term connectivity conservation. Together, these approaches identify priority areas and regions of uncertainty, providing a spatial framework to guide jaguar conservation and connectivity planning under accelerating global change.

## Graphical abstract

Using movement‐based connectivity models, we identify priority core areas and dispersal corridors for jaguars across the Americas, revealing strong connectivity in South America but major fragmentation in Mexico, Central America and the Atlantic Forest.

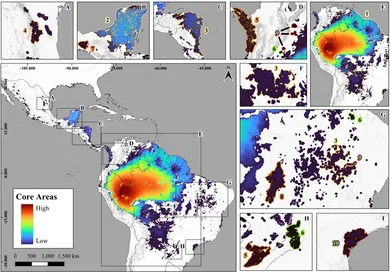

## 1 Introduction

The world has changed. Large portions of natural habitat have been transformed by anthropogenic actions (IPBES 2019), leading to the reduction and fragmentation of wildlife populations and—in the worst scenarios—even the extinction of species (Ceballos et al. 2015). The distribution of these anthropogenic impacts is not homogeneous, nor is the way in which they affect different taxa (Bardales et al. 2024; Cardillo et al. 2005). Large apex carnivores require extensive areas of undisturbed habitat and healthy prey populations to thrive (Sunquist and Sunquist 2002). Where apex carnivores remain, small population sizes and geographical isolation frequently limit successful dispersal processes, causing loss of genetic diversity (e.g., Srbek‐Araujo et al. 2018). In this context, landscape connectivity models are useful tools for predicting individual (or species‐level) movement across landscapes, identifying general patterns and informing conservation efforts (Taylor et al. 1993).

Landscape connectivity models have become increasingly central to conservation planning, providing spatially explicit and scientifically robust predictions of species movement that help prioritise conservation investments across entire ranges. As modelling techniques evolve and empirical data become increasingly available, researchers can now incorporate species' distribution, abundance and dispersal capacity into movement rates, generating more biologically realistic measures of connectivity (e.g., Cushman et al. 2013; Rudnick et al. 2012). These models typically identify two key landscape features: core areas—regions of high predicted movement density that support relatively stable wildlife populations and act as sources for dispersal—and corridors, which are landscape linkages facilitating movement between core areas. Such approaches have yielded important insights into carnivore ecology and conservation across diverse systems, revealing, for example, critical connectivity bottlenecks for tigers in Southeast Asia (Ash et al. 2026), dispersal corridors for bears in Iran (Mohammadi et al. 2021) and the role of unprotected lands in maintaining functional linkages for lions in Africa (Cushman et al. 2018). Furthermore, ranking both core areas and corridors by their relative contribution to range‐wide connectivity allows conservation efforts to be directed where they will have the greatest impact (Kaszta et al. 2019).

Jaguars ( *Panthera onca* ) have experienced significant range contraction over the last century (around 50%; Quigley et al. 2017). Although they still occupy an area of approximately 9 million km^2^, most populations outside the Amazon are isolated and highly threatened (de la Torre et al. 2018), with reduced genetic diversity already reported in some of them (Haag et al. 2010; Lorenzana et al. 2020). Protected areas have long been the cornerstone strategy for safeguarding ecological integrity and natural heritage (Rodríguez‐Rodríguez and Martínez‐Vega 2022). Indigenous lands have similarly proven effective in preserving natural habitats and their wildlife (Benítez‐López et al. 2019; Qin et al. 2023). Together, these land tenure categories—hereafter referred to collectively as PAs—safeguard large jaguar populations (Alvarenga et al. 2025; Bogoni et al. 2023) and extensive areas of suitable habitat (Alvarenga et al. 2026). However, a conservation strategy restricted solely to PAs is inadequate for large carnivores such as the jaguar, as PAs are often too small and too isolated due to changes in the surrounding landscape (Laurance et al. 2012), which limits their efficacy in maintaining viable populations. Therefore, as protected and unprotected jaguar populations are distributed across the landscape, maintaining connectivity among them is paramount to reduce the risk of local extinctions.

Several studies have assessed landscape connectivity across the jaguar range using an array of techniques and types of biological data, from expert knowledge (Rabinowitz and Zeller 2010) and interviews (Petracca et al. 2018) to empirical observations of jaguar occurrence (telemetry—de la Torre et al. 2017, camera traps—Meyer et al. 2020 and database compilations—Balbuena‐Serrano et al. 2022). However, due to limited data availability, only one study covered the entire jaguar range and was based solely on expert knowledge (Rabinowitz and Zeller 2010). Here, we present a broad‐scale modelling approach based on empirical telemetry data (Alvarenga et al. 2026) to assess jaguar population connectivity across its range, updating the previously available model (Rabinowitz and Zeller 2010). To support conservation prioritisation, we model and rank priority areas for the species (hereafter, core areas) and the corridors that connect them. In parallel, we extend this framework to PAs, ranking both PAs and their potential corridors, thereby providing quantitative information relevant to governments and international agencies.

## 2 Methods

### 2.1 Resistance Surface Modelling

Landscape connectivity models can be developed from different approaches (Unnithan Kumar et al. 2022), with resistance surface‐based methods being the most prominent (Zeller et al. 2012). Resistance surfaces represent the ‘physiological cost for a given species to move through a particular environment’ (Zeller et al. 2012). Therefore, low‐resistance values represent landscape units (pixels) more easily traversed by a focal species, whereas high‐values represent less permeable landscape units. We based our resistance surface modelling on a multi‐scale habitat suitability model developed across the historical range of jaguars, using empirical data from 172 GPS‐collared individuals (86 females and 86 males, *N* = 231,017 GPS locations; Table S1) (Alvarenga et al. 2026). Due to inconsistencies in data among individuals (mainly the insufficient frequency of telemetry fixes), the authors were unable to apply a movement model per se (e.g., Path Selection Function; Cushman and Lewis 2010). Instead, they applied a Point Selection Function with 80% of the GPS locations and the remaining 20% reserved for model validation (Boyce Index [*f*] = 0.91; AUC = 0.88). The fitted model showed that human population density, livestock density and human modification had the strongest negative effects on habitat selection, whereas soil cation exchange capacity, NDVI and proximity to water bodies exerted the strongest positive effects, indicating a preference for productive habitats located near water bodies.

To transform the habitat suitability surface into resistance to movement, we applied a negative exponential transformation (Keeley et al. 2016; Mateo‐Sánchez et al. 2015). This approach assumes that animals may move through suboptimal areas during long‐distance dispersal, a pattern previously reported for jaguars (Schoen et al. 2025), lions (Elliot et al. 2014), spotted hyenas (Beytell et al. 2024) and bears (Mateo‐Sánchez et al. 2014). The negative exponential transformation produces a resistance surface in which landscape resistance increases nonlinearly and convexly as suitability decreases, instead of increasing at a constant rate in a negative linear function. We applied the transformation function of Keeley et al. (2016), ‘*R* = 100 − 99 × ((1 − exp(−*c* × *h*))/(1 − exp(−c)))’, where ‘*R*’ is resistance, ‘*h*’ is the suitability layer, and ‘*c*’ determines the strength of the suitability‐to‐resistance transformation. A zero ‘*c*’ value means a linear transformation, and as ‘*c*’ increases, resistance values become an increasingly non‐linear negative exponential function of suitability. We evaluated a range of candidate ‘*c*’ values by comparing the resulting resistance surfaces against independently documented patterns of jaguar distribution and movement (Jȩdrzejewski et al. 2018; Schoen et al. 2025). Based on this assessment, we selected *c* = 16, which produced the most biologically realistic balance between maintaining low resistance across known jaguar strongholds and preserving contrast with less suitable habitats (see Supporting Information for details, Table S2, Figures S1–S4).

The resistance layer described above was used to calculate dispersal thresholds (see Section 2.3 for details). However, additional adjustments were required to account for landscape features that are either under‐represented or not explicitly captured by the habitat suitability model, and to avoid routing corridors through areas that are ecologically implausible. Such expert‐informed adjustments are widely used in large‐scale connectivity modelling to ensure that dispersal pathways derived from suitability layers remain biologically realistic (e.g., McRae et al. 2008; Penjor et al. 2024; Zeller et al. 2012). For example, although jaguars may occasionally move close to urban areas (G1 2019b), such events are sporadic and typically associated with high mortality risk (Bejarano Alegre et al. 2024). To minimise the likelihood of corridors being routed through urban centres, we applied a 4‐km buffer around the centroids of villages, towns and cities (OSM; www.openstreetmap.org), within which resistance values were increased by 1,000,000 cost units. The buffer width was defined as a practical compromise: while it may not entirely cover the extent of larger urban centres, it substantially reduces the chance of under‐representing urban influence while avoiding excessive coverage of smaller villages. Similarly, considering that jaguar records are extremely rare above 2500 m (Griffith et al. 2021), we applied a non‐linear transformation that increasingly penalised values above this threshold with resistance values rising to the cubic order. At a lower intensity, we also increased the resistance of roads (OSM; www.openstreetmap.org) and water bodies (Lehner and Grill 2013), acknowledging that jaguars are capable of crossing these features but that they can still impose movement costs (e.g., Hernández‐Pérez et al. 2020). For roads, we considered the three largest paved classes, where traffic volume and vehicle speed are highest, increasing the risk of jaguar mortality due to collisions. Accordingly, we added 10 cost units to motorways and trunk roads, and 5 cost units to primary roads. For rivers, we used water flow order as a proxy for crossing difficulty. The two highest‐order classes (orders 1 and 2), as well as lakes, were assigned an additional 10 cost units, while lower‐order rivers (orders 3 and 4) were assigned an additional 5 cost units. Finally, given that jaguars often use riparian forests for movement (Gese et al. 2018; Schoen et al. 2025), including in human‐modified landscapes and in proximity to settlements (G1 2019a), we adjusted resistance along major rivers using a land‐cover map (Brown et al. 2022). This adjustment was necessary because the habitat suitability layer, while capturing broad environmental gradients, tended to overestimate resistance in riparian environments embedded within otherwise high‐resistance matrices (e.g., near urban areas or agricultural mosaics). Land‐cover data were extracted from the 2023 Dynamic World collection, with the most frequent classification across the year assigned to each pixel. Riparian areas were defined as an 8‐km buffer on each river margin (orders 1–4), resulting in a 16‐km‐wide corridor—the scale shown to positively influence jaguar occurrence near water surfaces (Alvarenga et al. 2026). To capture decreasing resistance closer to riparian forests, we applied a linear decay based on river distance, reducing resistance by 50% in river‐adjacent forest pixels and gradually returning to the baseline at the buffer's outer edge (8 km) (Final Resistance layer: Figure S5).

### 2.2 Dispersal Source Points

To assess jaguar habitat connectivity, we applied two complementary modelling approaches: cumulative resistant kernels (Compton et al. 2007), which identify core areas of high predicted movement density, and factorial Least‐Cost Paths (fLCP) (Cushman et al. 2009), which model the narrow dispersal corridors linking these core areas. While resistant kernels capture broad patterns of movement intensity across the landscape, fLCPs are better suited to delineating the specific linkages between core areas; together, they provide a more complete picture of connectivity than either method alone. These models consider the rate of movement of organisms as a function of the species' distribution, abundance and dispersal capacity. Therefore, to define the distribution and density of jaguars across their current range, we used the previously mentioned habitat suitability layer (Alvarenga et al. 2026) combined with a jaguar population estimate layer (Jȩdrzejewski et al. 2018). Together, these layers provided a probabilistic surface from which source points, representing individual jaguars, were distributed across the landscape. First, we resampled the jaguar population layer to 500 m (same as the habitat suitability surface) and adjusted its pixel values to reflect potential jaguar density at a pixel level. Then, to reduce the number of candidate source locations while maintaining a density‐weighted spatial distribution, we implemented a stochastic filtering procedure. A uniform random raster (ranging from 0 to 1) was subtracted from the density raster, and only pixels with positive values were retained and transformed into source points (e.g., Macdonald et al. 2018). This approach ensured that the probability of retaining a pixel was proportional to its estimated jaguar density. The resulting source points were then subjected to a second filtering step to ensure that they were retained only in suitable habitat. The habitat suitability surface was divided into 10 quantiles, and source points falling within the five lowest quantiles were excluded. From the remaining points, we randomly selected 10,000 points to be used in the cumulative resistant kernels and 5500 points for the fLCP. These models focused on the linkages between landscapes that are biologically relevant for the species, regardless of their protection status.

As PAs are more likely to maintain natural habitats in the long term, we developed a second set of connectivity models using a different source‐point configuration. First, a centroid point was generated for each PA to ensure that every PA had at least one source point on it. Additional source points were then randomly sampled from the pool of source points that had passed both the density‐based and habitat suitability filtering steps, resulting in a total of 4500 source points. While this selection included a random component, larger PAs with high habitat suitability and predicted jaguar density had a higher likelihood of being assigned more points.

We used the same PA and jaguar current range layers as Alvarenga et al. (2026). PAs were obtained from two databases (Jȩdrzejewski, Morato, Negrões, et al. 2023; UNEP‐WCMC and IUCN 2022), retaining only those PAs (or contiguous blocks of PAs) larger than 100 km^2^ (Sollmann et al. 2008), located predominantly below 3000 m in elevation on average (Griffith et al. 2021), and intersecting the species' current range (*N* = 905 PAs, Figure S6). The jaguar's current range was also defined by combining two datasets: the South American distribution (Jȩdrzejewski, Morato, Wallace, et al. 2023) and the Wildlife Conservation Society jaguar distribution layer covering areas from Panama to the southern United States (Zeller 2007). To address spatial uncertainties and the species' high mobility, Alvarenga et al. (2026) applied a 100 km buffer around the estimated range, which was then used as the final extent of the jaguar's current distribution (Figure S6).

### 2.3 Connectivity Models

Due to computational constraints, we divided the study area into two regions (hereafter referred to as the northern and southern ranges), using the Panama Canal as a reference. This decision was partly based on Cushman et al. (2023), who predicted the canal to be an insurmountable barrier to large carnivores. We used UNIversal CORridor network simulator (UNICOR) (Landguth et al. 2012), applying two complementary methods: the cumulative resistant kernels (Compton et al. 2007) to define core areas of high‐density movement and fLCP to model dispersal corridors between these core areas (Cushman and Landguth 2012). UNICOR is a species connectivity identification tool that applies Dijkstra's shortest path algorithm to individual‐based simulations (Landguth et al. 2012).

Cumulative resistant kernels provide one of the most biologically realistic measures of comprehensive landscape connectivity, as they incorporate species' distribution, abundance and dispersal capacity into movement rates (Compton et al. 2007; Cushman et al. 2015; Unnithan Kumar and Cushman 2022). Resistant kernels have been found to perform well in predicting patterns of organism movement (Cushman et al. 2014; Lumia et al. 2024) and genetic diversity (Atzeni et al. 2024; Macdonald et al. 2018). This method does not assume a priori destinations but calculates connectivity across the entire landscape by measuring the functional distance from a source point to every other cell in the considered landscape (Compton et al. 2007). The model applies a least‐cost function to calculate the costs of moving from each source point to its neighbouring cells based on resistance surface values. The process continues as costs accumulate with each movement across neighbouring cells until a predefined cost‐distance threshold from the source is reached (Cushman and Landguth 2012). This threshold constrains the dispersal distance used in the connectivity analysis and is described in detail below. Then, all least‐cost kernels generated from all source points are summed to produce a cumulative resistant kernel surface whose values indicate the density of potential movement in every pixel (Compton et al. 2007; Cushman and Landguth 2012). Because least‐cost kernels tend to produce broadly circular patterns, reflecting movement expanding from a source point to all surrounding cells, we used this approach to identify core areas of intense movement. In contrast, fLCPs are better suited to identifying long, narrow linkages between core areas.

Habitat conditions and human pressure vary widely across the different biomes occupied by jaguars. Previous range‐wide habitat suitability assessments have shown that this heterogeneity can cause ecologically important regions to be overshadowed by the disproportionately large extent of continuous suitable habitat in the Amazon (Alvarenga et al. 2026). Therefore, considering the large heterogeneity found across our study area, we opted to take into account ecoregion differences when defining core areas and later during the ranking process (the ranking procedure is fully described in the following subsection). Ecoregions were derived from the classification of terrestrial biomes and ecoregions (Olson et al. 2001), but disjointed categories were treated separately, following the same approach as Antonelli et al. (2018) (Figure S7). Therefore, to define core areas from the cumulative resistant kernel surface, we (1) clipped the surface to the extent of the species' current range, (2) excluded zero values, then (3) excluded the lowest 10% of cumulative kernel values within each ecoregion (i.e., values below the 10th percentile), and (4) excluded any remaining individual area smaller than 100 km^2^. The rationale for selecting the 10th percentile threshold, together with a comparison of alternative thresholds, is presented in Figure S8.

To model the long‐distance dispersal corridors between the core jaguar habitats and PAs we used the fLCP approach. The fLCPs are an evolution of the regular Least‐Cost Paths and have the advantage of being capable of calculating narrow landscape linkages (corridors) among many source‐destination pairs (Cushman et al. 2009; Cushman and Landguth 2012). Therefore, using the resistance layer as a basis, we calculated least‐cost paths from each jaguar source point to every other source point within a predefined dispersal cost distance (dispersal thresholds detailed below). The final corridors surface is generated by summing all least‐cost paths; thus, it represents the number of cost paths crossing each cell, which indicates the corridor strength (Rudnick et al. 2012). Since this final summed fLCP layer represents only narrow pixel‐wide linkages, we applied a smoothing moving‐window filter with an arbitrarily selected width of 9 km to improve the interpretability of the maps and GIS layers at the range‐wide scale. Using both connectivity modelling methods allows us to map, quantify and prioritise key areas and corridors that are most important for jaguar population connectivity (e.g., Cushman et al. 2018).

Our study area spans an extensive and heterogeneous region, resulting in significant variation in the resistance surface. This variability makes it challenging to establish a reliable dispersal threshold. Moreover, to the best of our knowledge, robust empirical estimates of jaguar dispersal distances are largely lacking in the scientific literature (see review in Gonzalez‐Borrajo et al. 2017), with only sparse and disparate reports available, ranging from relatively short movements (~50 km; Morato et al. 2016) to exceptional long‐distance dispersal events exceeding 2100 km (Morant et al. 2026). Therefore, to define the resistant cumulative kernel threshold, we selected 1,000,000 cost units, representing a conservative estimate of short‐distance movements. This value was calculated by multiplying the shortest documented movement (~50 km) by the mean resistance of the resistance surface (21.4 cost units). For long‐distance dispersal movements modelled through the fLCP corridors, we relied on the estimates from Bowman et al. (2002), which suggest that the median dispersal distance of mammalian species is isometrically related to their home‐range size by a factor of seven. So, we selected the second largest home range observed (HR = 2534 km^2^) among 111 collared jaguars that presented range residency (Thompson et al. 2021). We deliberately avoided basing the dispersal threshold on a single extreme home‐range estimate because it could disproportionately influence the resulting threshold. Then, we calculated the square root of this value and multiplied it by seven to estimate the median potential dispersal distance jaguars could achieve, resulting in 352 km. Assuming the same mean resistance value (21.4 cost units), this corresponds to approximately 7,500,000 cost units (352 km × 21.4 cost units), which was used as the threshold for the fLCP analysis. While movement patterns are expected to vary among jaguar populations inhabiting different habitats (Morato et al. 2018), quantifying such differences remains challenging given the available data; therefore, we assumed uniform dispersal capacity across the species' range.

### 2.4 Identifying and Ranking Core Areas, Protected Areas and Corridors

To identify the most important core areas, PAs and their associated corridors, we ranked them using independent metrics, following the approach of previous studies (e.g., Cushman et al. 2018; Kaszta et al. 2019). Core areas and PAs were first ranked independently by (1) their size and (2) the sum of kernel density values within their extent. These two ranks were then averaged, so that core areas/PAs that were both large and contained high kernel sums received the highest scores. To account for regional variation across the jaguar range, this average ranking was subsequently recalculated within each ecoregion. Thus, a core area or PA ranked third overall could become the highest‐ranked feature within its ecoregion if no other feature achieved a higher average score in that ecoregion. Finally, for core areas/PAs spanning multiple ecoregions, we calculated the arithmetic mean of their rank positions across all overlapping ecoregions.

To calculate the metrics used to rank our two sets of corridors (between core areas and between PAs), we first used the narrow, pixel‐wide linkages derived from the summed fLCP layers. Each corridor was independently ranked according to three criteria: (1) the total LCP values within its extent, (2) the number of core areas or PAs it connected (excluding corridors linking the same area), and (3) the mean kernel value of the connected core areas or PAs. These three rankings were averaged to produce an initial composite rank. Next, we applied the wide‐version corridors (9 km), which merged nearby corridors into larger networks. For these merged networks, we recalculated rankings by averaging the positions of the component corridors, weighted by their size so that larger networks connecting more areas contributed proportionally more to the rank. Finally, this combined ranking was reassessed at the ecoregion scale using the same approach as described for core areas and PAs.

## 3 Results

### 3.1 Core Areas Ranking

**FIGURE 1:**
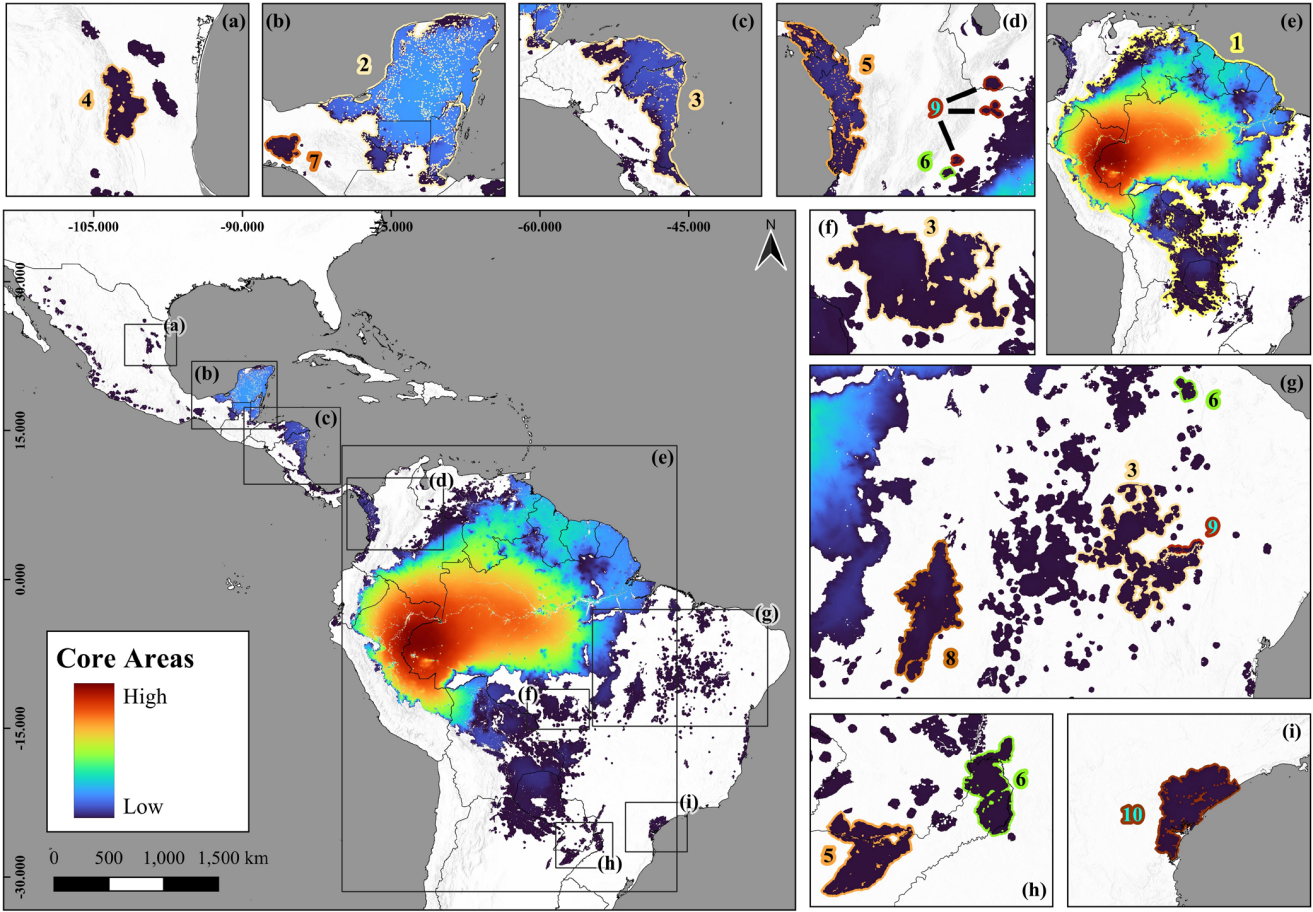
Ranking of core habitat areas for jaguars across the species' current range. The strength of core areas is expressed as density of movement. The side maps highlight the 10 highest‐ranked core areas numbered by priority. In the side maps, buffer colours correspond to the respective rank positions. Map lines delineate study areas and do not necessarily depict accepted national boundaries.

Based on the cumulative resistant kernel analysis, we identified 307 core areas for jaguar movement, representing regions of high predicted movement density: 70 in the northern range and 237 in the southern range (Figure 1). Core areas cover approximately 7.75 million km^2^, representing 55% of the jaguar's current range, with about half of this area under formal protection. The largest and highest‐ranked core area spans 6.5 million km^2^, encompassing four biomes—Llanos, Amazon, Chaco and Pantanal—and 11 countries (Figure 1e, Table 1). The Mayan Forest ranked second (Figure 1b), followed in third place by three areas of equal rank: the Central American Moist Forest, the southern Amazon, and the Cerrado‐Caatinga transition zone (Figure 1c,f,g). The full ranking is provided in Alvarenga et al. (2026), Zenodo dataset. The top‐ranked core area alone accounts for 85.5% of the total predicted core area and 98.6% of cumulative kernel values. Considering the five highest‐ranked areas together, these proportions increase to 91.4% of the total area and 99.9% of kernel values. Nonetheless, the ecoregion‐stratified approach also highlighted smaller core areas of regional importance, several of which appear among the 10 highest‐ranked (Figure 1a–i, Table 1).

**TABLE 1:** Ten highest‐rank core habitat areas for jaguars across the species' current range, showing the countries encompassed by each core area, total area (km^2^), kernel sum and final rank. Final rank positions (1–10) are also shown spatially in the side maps of Figure 1.

Countries	Area (km^2^)	Kernel sum	Priority rank
Argentina, Bolivia, Brazil, Colombia, Ecuador, Guyana, French Guyana, Paraguay, Peru, Suriname, Venezuela	6,540,315	2514.70	1
Belize, Guatemala, Mexico	171,255	26.49	2
Costa Rica, Honduras, Nicaragua	95,619	5.31	3
Brazil	78,215	0.10	3
Brazil	94,395	0.40	3
Mexico	9134	0.03	4
Colombia, Panama	65,458	1.69	5
Argentina, Paraguay	21,013	0.02	5
Colombia	1209	< 0.01	6
Brazil	3813	< 0.01	6
Argentina, Brazil, Paraguay	15,954	0.01	6
Mexico	7524	0.03	7
Brazil	62,606	0.27	8
Colombia, Venezuela	2308	< 0.00	9
Colombia	1866	< 0.00	9
Colombia	875	< 0.00	9
Brazil	2050	< 0.00	9
Brazil	23,509	0.08	10

### 3.2 Corridor Ranking

**FIGURE 2:**
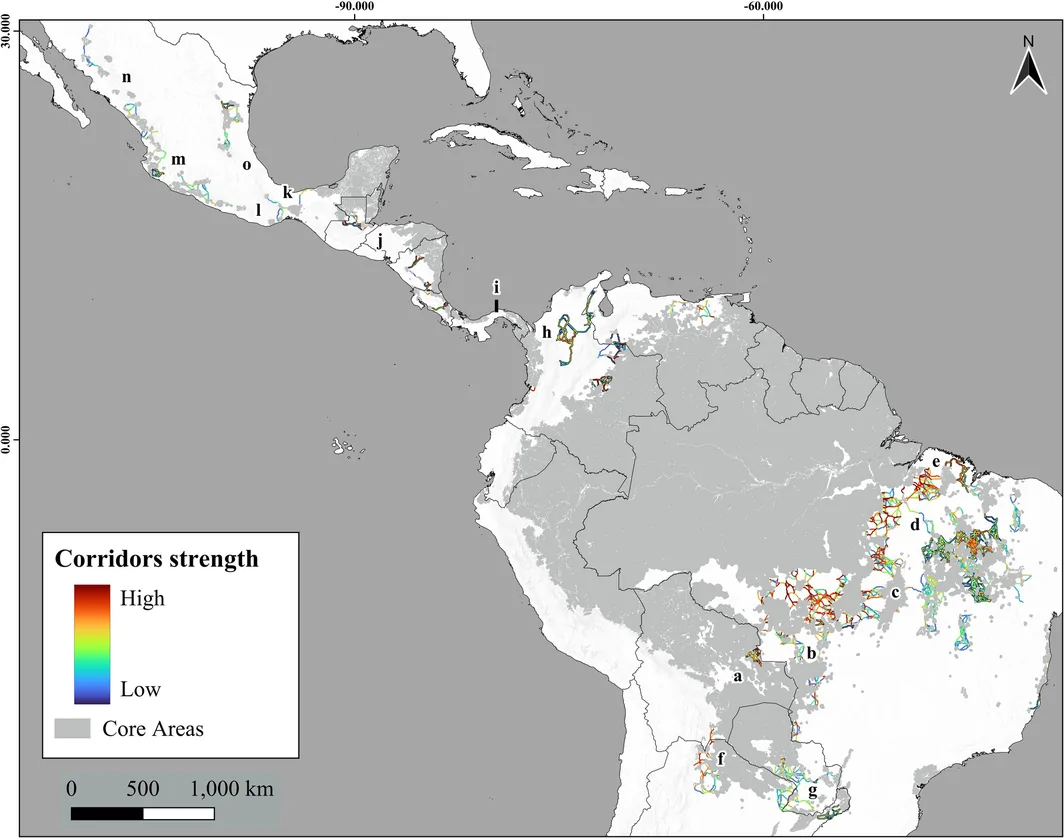
Corridors' strength between core habitat areas for jaguars across the species' current range. Letters represent areas of interest described in detail in Section 4. Map lines delineate study areas and do not necessarily depict accepted national boundaries.

**FIGURE 3:**
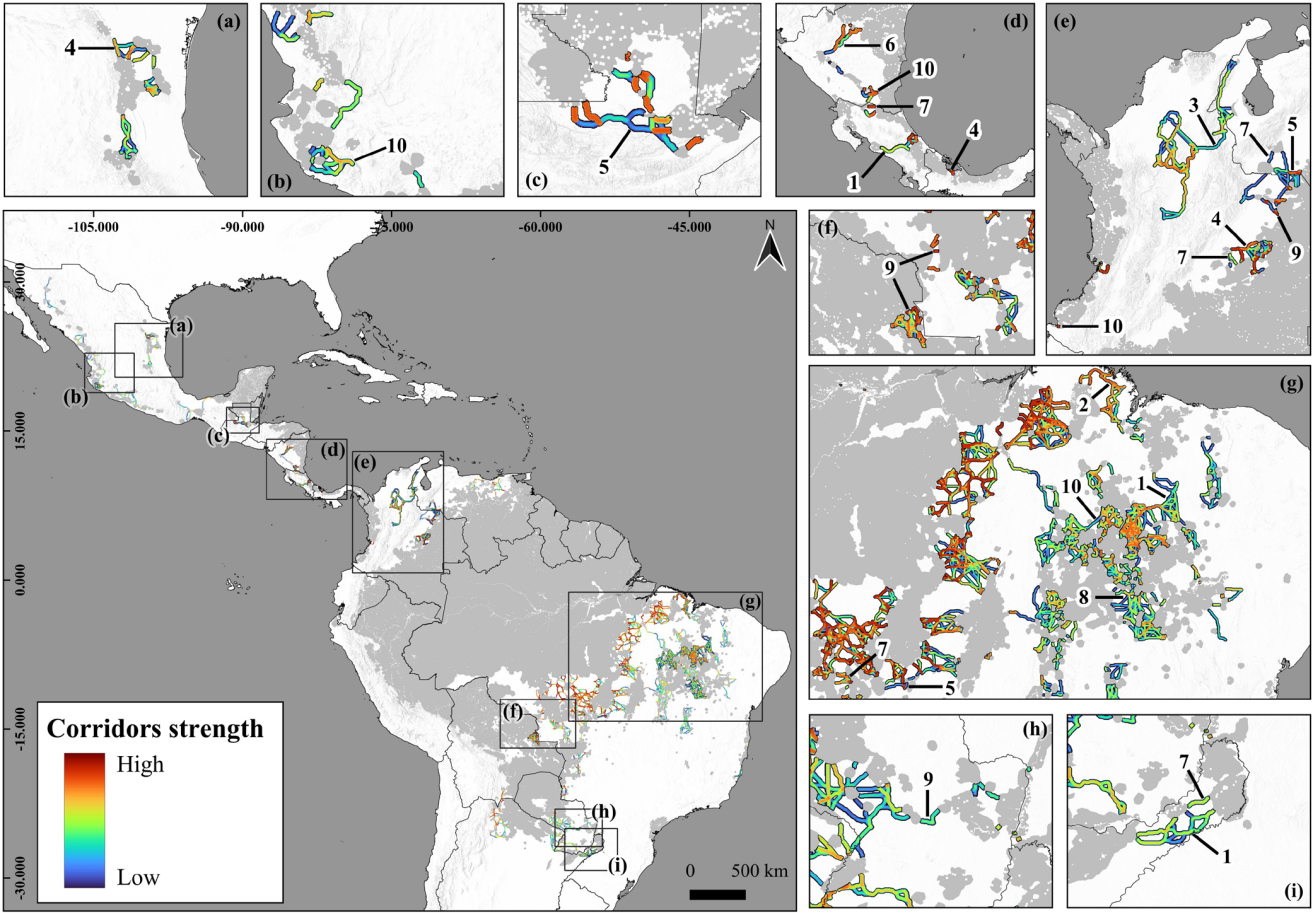
Ranking of the corridor's strength between jaguar core habitat areas across the species range (a–i). Numbers represent rank prioritisation. Map lines delineate study areas and do not necessarily depict accepted national boundaries.

We identified 176 fLCP dispersal corridors connecting jaguar core habitat areas and ranked them by strength (Figure 2; full data available in Alvarenga 2026). Of these, 45 corridors were located in the northern range and 131 in the southern range. However, our models did not indicate connectivity across the entire species' range. In the northern range, Mexico contains four interlinked groups of core areas, but these do not connect with each other or with the Mayan Forest to the south (Figure 3). A similar pattern emerges in Central America, where three major interconnected groups remain isolated: the Mayan Forest; a large cluster spanning Honduras to Panama; and the Darién Gap on the Panama‐Colombia border. Notably, all three contain corridors ranked among the 10 strongest (Figure 3c,d, Table 2). In South America, our ranking highlights an important corridor (third highest overall) extending from the Venezuela–Colombia border towards the Darién Gap. However, this corridor does not connect to the Gap or to the largest southern core area, apparently blocked by the northern end of the Andes (Figure 3e). The highest‐ranked core area—Llanos/Amazon/Chaco/Pantanal—shows multiple connections with smaller areas, suggesting links to the Cerrado and Caatinga biomes in north‐eastern Brazil, as well as to Iberá and the Green Corridor of the Atlantic Forest in Argentina and Brazil at the species' southern range limit. This represents the largest and most significant interlinked network of core areas, with several corridors among the 10 highest‐ranked (Figure 3e–i). In contrast, all coastal Atlantic Forest patches appear isolated from this group and from each other, including the *Serra do Mar* core area in Brazil (Figure 3).

**TABLE 2:** Ten highest‐rank corridors between jaguar core areas across the species' current range, showing the countries encompassed by each corridor, total area (km^2^), IDs of narrow fLCP corridors, least‐cost path sum of these corridors, kernel mean of the core areas linked and final rank. Final rank positions (1–10) are also shown spatially in Figure 3.

ID	Area (km^2^)	Country	fLCP_IDs	LCP sum	Kernel mean	Priority rank
13	28,617	Brazil	93	73,119	0.04	1
72	3920	Argentina	263	2275	0.01	1
133	1757	Costa Rica	56	26,340	1.78	1
28	8590	Brazil	77, 83	40,510	0.06	2
128	20,311	Colombia, Venezuela	51	23,072	0.00	3
125	5807	Colombia	68	668,669	838.23	4
132	259	Panama	60	11,448	0.02	4
153	1820	Mexico	5	2811	0.01	4
68	1064	Brazil	194	21,535	0.01	5
122	5443	Colombia, Venezuela	63, 66, 67	100,885	1047.79	5
142	2700	Guatemala	42	17,802	8.83	5
137	2123	Nicaragua	45	10,623	1.77	6
2	1525	Brazil	228	814	0.00	7
74	435	Argentina	262	350	0.01	7
84	1626	Brazil	188	19,446	0.20	7
124	371	Venezuela	62	40	0.00	7
127	414	Colombia	69	249	838.23	7
136	220	Costa Rica	50	11,520	2.66	7
14	16,369	Brazil	141, 142	12,070	0.04	8
91	457	Paraguay	255	165	0.00	9
108	143	Brazil	191	23,952	1257.55	9
110	5995	Bolivia	216, 218	224,757	419.12	9
123	828	Colombia	65	86,603	1257.35	9
30	22,126	Brazil	101, 103, 107, 111, 114	20,599	0.03	10
131	74	Colombia	73	4656	0.05	10
134	1048	Nicaragua	48, 49	8434	0.67	10
167	2460	Mexico	18, 19, 20, 21	1841	0.00	10

### 3.3 Ranking Protected Areas and Their Corridors

**FIGURE 4:**
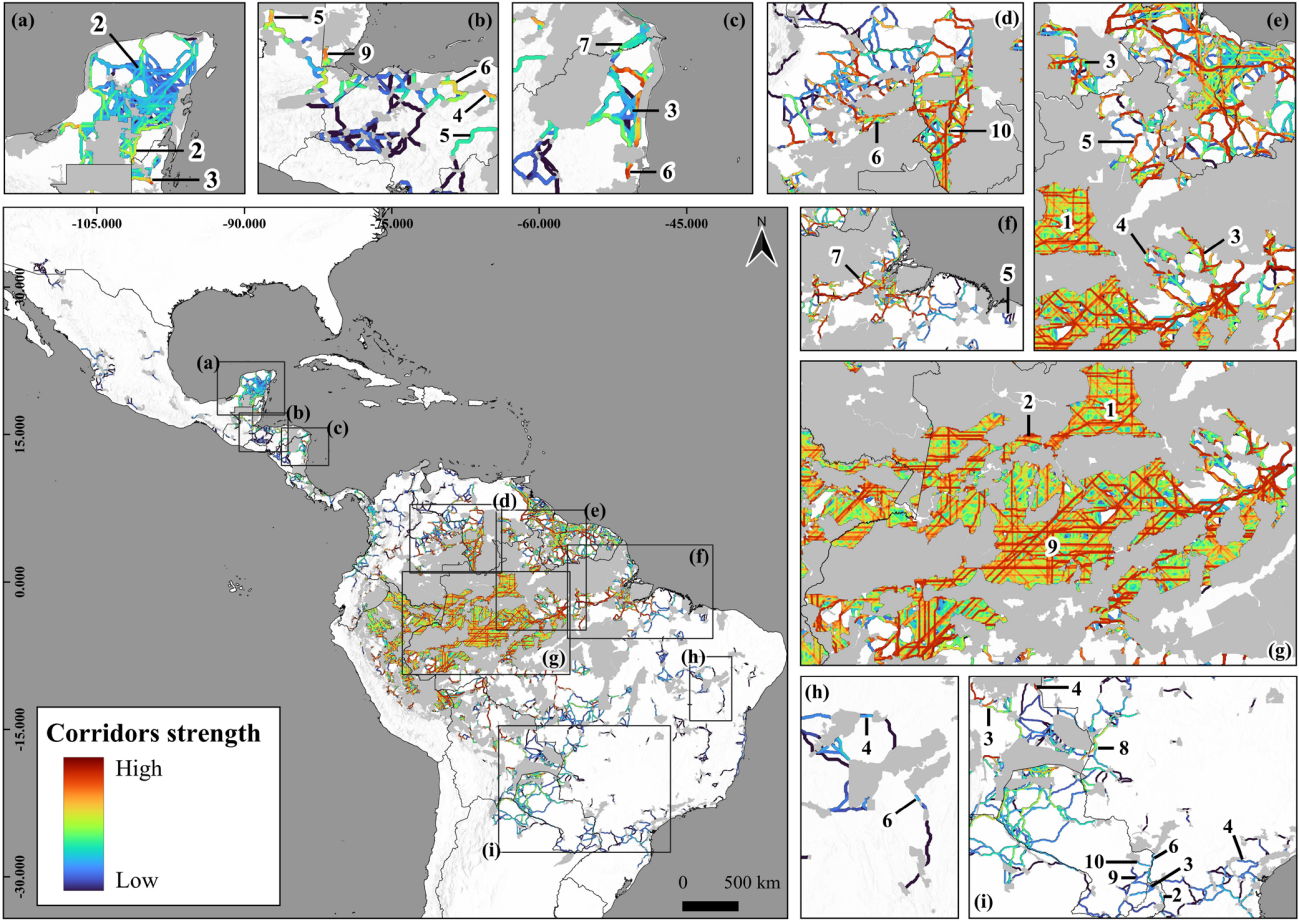
Ranking of the corridor's strength among protected areas and indigenous lands (PAs) across the species current range (a–i). Numbers represent rank prioritisation. Map lines delineate study areas and do not necessarily depict accepted national boundaries.

**FIGURE 5:**
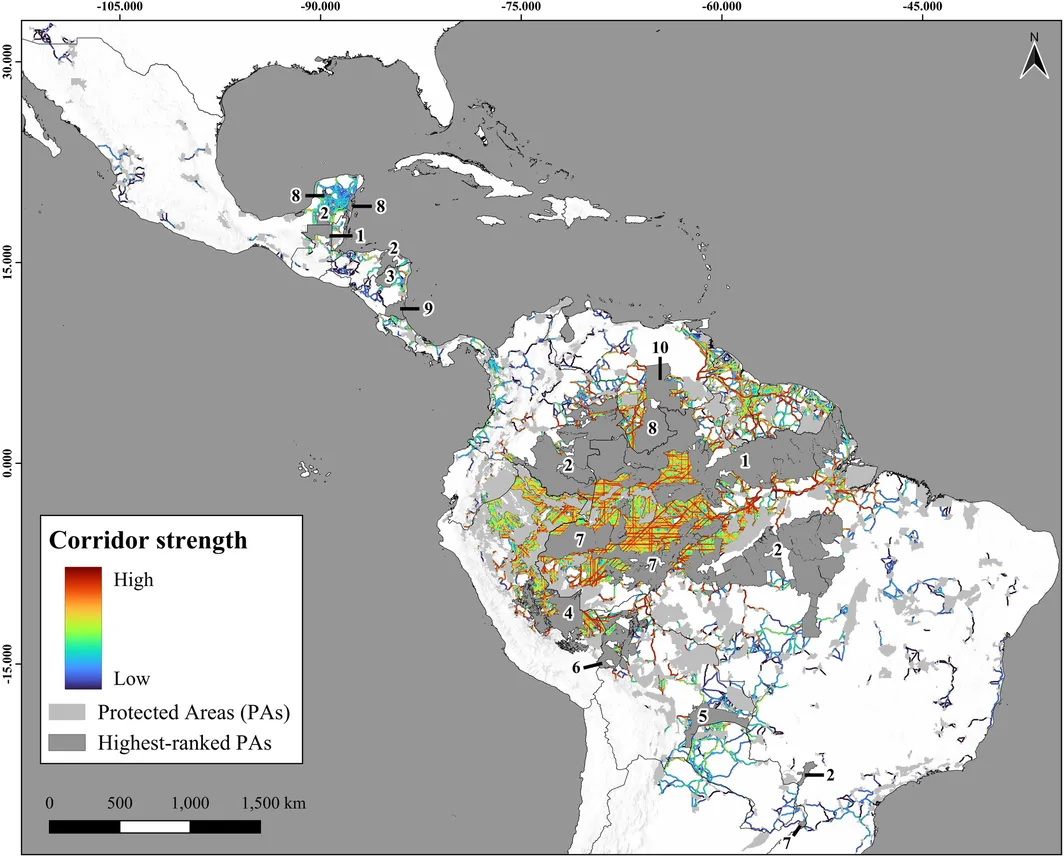
Jaguar corridor network among protected areas and indigenous lands (PAs) across the species' current range. Numbered PAs represent the 10 top‐ranked ones. Map lines delineate study areas and do not necessarily depict accepted national boundaries.

In the alternative approach, we predicted potential jaguar corridors between PAs, ranking both the PAs and their associated corridors. In total, 507 fLCP corridors were identified among 905 PAs: 103 corridors in the northern range and 404 in the southern range (Figures 4 and 5; full data available in Alvarenga 2026). In the northern range, Mexico showed a broadly similar pattern to the previous approach, with clusters of PAs interlinked in roughly the same locations as the clusters of connectivity identified among core areas and corridors—in both cases such clusters appeared isolated from the rest of the network. Moving south into Central America, however, predictions indicated improved connectivity compared with the previous approach, as corridors linked PAs from the Mayan Forest to Panama; several of these corridors ranked among the top 10 (Figure 4a–c). Nevertheless, the connection gap between this long corridor network and the Darién Gap persists. In South America, predictions were more positive, revealing a potential corridor from the Darién Gap through northern Colombia to the Venezuelan border, where it links to the Llanos and Amazon (Figures 4 and 5). This extensive PA‐corridor network extends across the Amazon biome and southward to the Chaco, Pantanal, Iberá, the Green Corridor and—surprisingly—the *Serra do Mar* in the Brazilian Atlantic Forest. Several of the highest‐ranked corridors occur within this network (Figure 4d–i, Table 3). Worryingly, the Cerrado and Caatinga biomes lost their connection to the wider network under this approach, and the northern Atlantic Forest patches remained isolated. Regarding PA ranking (Figure 5, Table 4), a strong concentration of high‐ranked areas occurred in the Amazon biome, consistent with their extensive size and high‐quality habitat. At the same time, the ecoregion‐stratified strategy used in this study ensured that top‐ranked PAs were also represented more broadly, spanning the Mayan Forest, Central America, the Chaco and the Atlantic Forest (Table 4; full data available in Alvarenga 2026).

**TABLE 3:** Ten highest‐rank corridors between protected areas and indigenous lands (PAs) across jaguar current range, showing the countries encompassed by each corridor, total area (km^2^), IDs of narrow fLCP corridors, least‐cost path sum of these corridors, kernel mean of the PAs linked and final rank. Final rank positions (1–10) are also shown spatially in Figure 4.

ID	Area (km^2^)	Country	fLCPs_IDs	LCP sum	Kernel mean	Priority rank
207	62,510.7	Brazil	617, 769, 772	96,452,202	165.44	1
124	424.5	Argentina	402	1600	< 0.01	2
247	7290.4	Brazil	800	18,185,617	165.44	2
435	44,706.7	Mexico	141, 202, 207	913,443	2.41	2
450	3058.2	Belize, Mexico	178	229,973	2.06	2
129	1815.3	Argentina, Paraguay	424	2177	< 0.01	3
159	6893.1	Brazil	495	9,488,060	159.76	3
208	14,005.0	Venezuela	619, 678	17,064,436	65.99	3
223	1384.7	Bolivia	657	240,891	1.89	3
410	7966.1	Nicaragua	70	990,641	0.44	3
447	1501.0	Belize	172	194,146	1.37	3
31	351.4	Brazil	2243	576	0.01	4
82	1023.1	Brazil	242	605	0.02	4
169	3517.5	Brazil	521	4,709,562	159.76	4
194	300.8	Bolivia	585	182,595	0.60	4
424	336.1	Honduras	124	46,907	0.51	4
36	2331.7	Brazil	2229	757	< 0.01	5
177	14,740.8	Brazil, Guyana	537, 606	12,021,921	102.39	5
423	1208.6	Honduras	123	15,755	0.76	5
458	815.9	Guatemala	206	78,410	1.56	5
21	156.6	Brazil	2240	256	0.02	6
130	655.1	Brazil, Paraguay	423	1460	< 0.01	6
276	6096.3	Colombia	1036, 1128	4,750,364	84.75	6
412	350.8	Nicaragua	76	167,837	0.18	6
425	1135.4	Honduras	125	80,275	< 0.01	6
68	82,664.8	Brazil	232, 233, 260, 277	219,293,265	21.29	7
411	2344.7	Honduras, Nicaragua	72, 73	43,661	0.48	7
147	3705.2	Brazil	464	174,099	0.02	8
131	2824.9	Brazil, Paraguay	429, 433	5247	< 0.01	9
154	417,269.7	Brazil	478, 562, 587, 653, 656, 670, 684, 687, 698, 752, 753, 754, 756, 757, 758	972,294,324	92.62	9
452	518.1	Belize	182	98,200	0.28	9
132	637.1	Paraguay	432	1168	< 0.01	10
244	91,927.0	Colombia, Venezuela	786, 810, 813, 819, 977, 984, 1033, 1271, 1301	84,293,863	11.35	10

**TABLE 4:** Ten highest‐rank protected areas (PAs) across jaguar current range, showing the countries encompassed by each PA, total area (km^2^), kernel sum and final rank. Final rank positions (1–10) are also shown spatially in Figure 5.

ID	Country	Area (km^2^)	Kernel sum	Priority rank
133	Brazil	753,553	317.13	1
545	Guatemala	22,340	3.10	1
487	Colombia	321,233	169.45	2
246	Brazil	495,192	131.68	2
621	Mexico	14,095	2.85	2
598	Honduras	19,333	1.51	2
345	Brazil	10,084	0.00	2
682	Nicaragua	19,832	0.94	3
811	Peru	141,428	80.67	4
52	Bolivia	64,558	1.40	5
32	Bolivia	89,894	21.06	6
60	Brazil	142,890	131.38	7
211	Brazil	180,574	123.26	7
2	Argentina	2259	0.00	7
839	Venezuela	84,003	38.62	8
661	Mexico	6242	1.08	8
663	Mexico	1367	0.26	8
680	Nicaragua	17,429	0.23	9
879	Venezuela	77,540	24.09	10

## 4 Discussion

Maintaining and restoring ecological connectivity has become a global conservation priority to halt biodiversity loss and enhance ecosystem resilience, as recognised in the Kunming‐Montreal Global Biodiversity Framework (CBD 2022). Human‐driven land transformation has degraded approximately 75% of Earth's terrestrial surface, increasingly fragmenting natural habitats and isolating populations worldwide (IPBES 2019). In this context, our model revealed a central core area within the jaguar's range, spanning portions of the Llanos, Amazon, Chaco and Pantanal biomes (Figure 1). This region supports high levels of movement and connectivity, representing a cornerstone for the species' long‐term persistence. Nevertheless, regardless of the modelling approach used—whether linking core areas or PAs—major connectivity gaps persist across the jaguar's range (Figures 2, 3, 4). This pattern reflects a broader global trend, whereby anthropogenic landscape modification reduces long‐distance movements of terrestrial mammals (Tucker et al. 2018), progressively restricting connectivity and increasing isolation. Such isolation can reduce gene flow and genetic diversity, with particularly strong effects documented among large‐bodied mammals (Lino et al. 2019). Beyond individual populations, the disruption and loss of animal movement can have cascading ecological consequences by altering species interactions and ecological processes, including seed dispersal and other ecosystem functions (Dirzo et al. 2014).

Evidence of increasing genetic isolation has already been documented in jaguar populations across the Atlantic Forest and Mesoamerica (Srbek‐Araujo et al. 2018; Wultsch et al. 2016). Despite these signs of increasing isolation, jaguars remain in a comparatively favourable position among large carnivores. Unlike many other species whose distributions have become severely fragmented following extensive habitat loss—including range contractions of 99% for red wolves ( *Canis rufus* ), 95% for tigers ( *Panthera tigris* ) and 94% for lions ( *Panthera leo* ) (Wolf and Ripple 2017)—jaguars still retain a vast and largely connected core area centred on the Amazon Basin. This region likely represents one of the last opportunities to maintain continental‐scale connectivity for a large felid. At the same time, our results demonstrate that this stronghold alone is insufficient to ensure connectivity throughout the species' range. The persistent gaps identified outside the Amazon therefore reinforce the urgency of protecting remaining movement corridors and restoring connectivity in the face of accelerating global change.

### 4.1 Core Areas Connectivity

The ranking of core areas highlights the jaguar's reliance on a limited number of extensive and interconnected strongholds, especially within the central portion of its range. The dominance of the Llanos‐Amazon‐Chaco‐Pantanal complex in the ranking confirms this region as the backbone of continental connectivity, consistent with previous work identifying it as the species' demographic core (Jȩdrzejewski, Morato, Wallace, et al. 2023). However, habitat quality is not homogeneous across this vast area (Alvarenga et al. 2026; this study), and the region of central Bolivia, where the northern (Llanos‐Amazon) and southern (Chaco‐Pantanal) sections converge (Figure 2a), warrants particular attention, as the connection there is eroding with deforestation and wildfires being the main drivers of habitat loss (Insight Crime and Iguarapé Institute 2024). Moreover, genetic studies suggest limited connectivity between these two sections (Lorenzana et al. 2020; Roques et al. 2016). As acknowledged by the authors, though, limited sample size and its uneven geographic distribution may have influenced these patterns (Lorenzana et al. 2020; Roques et al. 2016). In both studies, samples were collected solely in southern Pantanal with none from the northern‐southern transition zone in central Bolivia. Targeted actions are therefore needed to investigate the effectiveness of this link and, if confirmed, to maintain natural habitats that safeguard dispersal within the central core.

Our fLCP analysis also indicated two other potential alternative corridors between the northern and southern sections within Brazil, both starting at the northern edge of the Pantanal (Figures 2b and 3f). One corridor follows the Paraguay River, while the other extends eastward near *Chapada dos Guimarães* National Park. From these starting points, both corridors continue north through small clusters of core areas until reaching the southern limit of the Amazon main core. These corridors cross several major roads and the Arc of Deforestation, yet they still coincide with known jaguar populations along their extent (Moraes 2012). Moreover, as both routes lie entirely within Brazil, coordinated national efforts could facilitate their protection and restoration.

Along the eastern Amazon borders, our model also detected potential corridors linking the central core to two other regions: the Araguaia River plains and the clusters of core areas in the Cerrado‐Caatinga interface (Figures 2c–e and 3g). The Araguaia River plains are likely the most strategic corridor connecting the central jaguar populations of the Cerrado to the Amazon (Moraes 2012). These plains harbour key jaguar populations across nearly 20 PAs along their margins (Silveira 2004). Their potential as a corridor has been suggested previously; however, the high level of anthropogenic disturbance in their central and northern portions could pose a major impediment to movement (Silveira et al. 2014). This intense disturbance may have disrupted the direct connection, leading our models to identify an alternative route between the Araguaia River plains and the Amazon core, formed by three independent networks of corridors that extend westward towards the Xingu complex (Figures 2c and 3g). All corridors in this region traverse large sections of the Arc of Deforestation, where only small forest fragments remain, with many of them lacking formal protection (MapBiomas Project 2025).

The same pattern applies to the connections linking the Amazon to the Cerrado‐Caatinga cluster (Figure 2c–e). Our model revealed a few potential links: one direct corridor following the Tocantins River (Figure 2d), and two others using intermediate stepping stones—one through the Araguaia River plains (Figure 2c) and another through isolated Amazonian fragments in the state of Maranhão, Brazil (Figure 2e). Similar routes have been identified in previous studies (Jȩdrzejewski, Morato, Wallace, et al. 2023; Silveira et al. 2014) but, as noted earlier, the landscape across this region is highly transformed, which could lead to limited dispersal movements and promote isolation, as genetic analyses suggest at least for the Caatinga population (Lazzari et al. 2025; Roques et al. 2016). In addition, recent estimates indicate that the Cerrado‐Caatinga cluster continues to experience high rates of habitat loss, posing a serious threat to an already reduced population (Thompson et al. 2023). Considering that this cluster represents one of the last refuges for a singular jaguar population adapted to the karstic conditions of the region, we emphasise the need for urgent conservation action in the area.

The southern border of the central core extends across Chaco in central Paraguay and northern Argentina, and our model identified potential corridors linking the central core to other small core areas in its western and eastern edges (Figure 2f,g). To the west, the predicted corridors are relatively short, connecting the Chaco portion of the central core to the Yungas population along the eastern slopes of the Andes, as well as to a few small core areas in the southwest (Figure 2f), where permanent jaguar populations are unlikely to persist (De Bustos et al. 2024). This Chaco‐Yungas connection, although short, appears uncertain and likely weak, as jaguar records from the Argentine Chaco are scarce and the population faces a high risk of extinction (Bitetti et al. 2016; Quiroga et al. 2014). Density estimates in the Yungas region also indicate low jaguar numbers (Reppucci et al. 2024); nevertheless, it still supports the largest remaining population in Argentina (Bitetti et al. 2016) and there is evidence of ongoing connectivity with its western edge (Caruso et al. 2023).

The jaguar core areas identified by our model in the southern range closely align with recent predictions of the species' distribution (Thompson et al. 2023), but both models may be somewhat overoptimistic, particularly in the central Chaco region, where permanent occupation seems increasingly sparse (Bitetti et al. 2016; Romero‐Muñoz et al. 2025). That said, the eastern corridors predicted by our model suggest potential links between the central Chaco and the Upper Paraná Atlantic Forest across Argentina, Paraguay and Brazil (Figures 2g and 3h,i). These corridors follow two independent routes: one through central‐southern Paraguay and another through Iberá in Argentina, both ultimately converging in the Green Corridor. While jaguar populations in Iberá and in the Green Corridor are recovering thanks to strong conservation efforts implemented by Fundación Rewilding Argentina (Donadio et al. 2022) and *Projeto Onças do Iguaçu*, their connections with the Chaco portion of the central core remain untested. According to our connectivity model among core areas, the Green Corridor represents the last remnant of Atlantic Forest still potentially connected to the central core, suggesting that all other populations in the biome are now isolated—a worrisome pattern, as genetic drift and reduced diversity have already been reported for these populations (Lorenzana et al. 2020; Srbek‐Araujo et al. 2018). Such isolation reflects the intense landscape conversion across the region, compounded by high human population density and a dense network of roads, all of which directly or indirectly contribute to jaguar mortality and displacement (Alegre et al. 2025; Cerqueira et al. 2021; Paviolo et al. 2016). For these regions, integrated management actions aimed at restoring genetic connectivity—through the management of individuals among isolated populations—remain the only plausible long‐term conservation strategy for the species (Srbek‐Araujo et al. 2018).

Moving north beyond the central core, across Colombia and throughout Central America, similar patterns of extensive habitat conversion associated with high human densities and road development once again disrupt connectivity across multiple regions (Figures 2h–j and 3c–e). Along the Venezuela–Colombia border, our models identified a narrow strip of corridors linking small core areas across the boundary and extending along the Magdalena River valley in north‐central Colombia (Figure 2h). This is an important region, as it suggests potential connectivity between known jaguar populations in the valley and areas of high conservation priority, such as the *Serranía de San Lucas*, where a new PA has been proposed (Boron et al. 2016; Machado‐Aguilera et al. 2024; Payán et al. 2016). However, our model also indicates that these populations are likely isolated by the rugged topography of the Andes, which appear to hinder connectivity with the central core to the south and the Darién Gap to the north.

From the Darién Gap on the Colombia‐Panama border to the Mayan Forest in Guatemala‐Belize‐Mexico, three main core areas were identified, separated by two major connectivity gaps: one in central Panama and another between Guatemala and Honduras (Figure 2i,j). The distribution and extent of these areas closely align with previous findings (Calderón et al. 2022), reinforcing their role as strongholds for the species in the region. Nevertheless, the Nicaraguan core area has experienced rapid agricultural expansion, even within protected areas, which appears to have reduced its suitability for jaguars (Petracca et al. 2014). In addition, these three core areas are highly susceptible to clandestine environmental degradation linked to narcotrafficking activities (Magliocca et al. 2024), which contribute to habitat fragmentation (Tellman et al. 2020) and the illegal trade of jaguar parts (Arias et al. 2020).

Regarding the two connectivity gaps, although Rabinowitz and Zeller (2010) predicted potential links in both areas, later assessments based on interviews (Petracca et al. 2018) and genetic analyses (Calderón et al. 2022; Wultsch et al. 2016) indicate that the corridor between Guatemala and Honduras is particularly weak. These gaps in connectivity across Colombia and Central America are pivotal for jaguar conservation, as they will determine whether northern and southern populations remain part of a continuous metapopulation. Implementing corridors in this region, however, poses substantial challenges, given that they extend across several countries and are impacted by multiple human disturbances. Creative and context‐specific conservation strategies will therefore be essential. The proposed protected area in *Serranía de San Lucas*, for instance, will be the first in Colombia to incorporate an Integrated Management District, promoting sustainable agriculture and mining within its buffer zone. If effectively regulated, this approach could represent a promising model for reconciling local development with the maintenance of natural habitats and may offer a transferable framework for other highly human‐modified regions, such as Central America.

The pattern of fragmented connectivity continues northward from the Mayan Forest towards Mexico's Gulf and Pacific coasts, where several gaps separate clusters of core areas (Figure 2k–o). This contrasts with previous models suggesting continuous suitable habitat and corridors extending along both coasts and converging at the Mayan Forest (Ceballos et al. 2021; Rodríguez‐Soto et al. 2013). Such differences may stem from variations in the biological data and analytical approaches; for instance, contrary to the previous studies, our models imposed a dispersal threshold that limits how far corridors extend according to landscape resistance. Overall, the connectivity gaps we identified coincide with regions of high human footprint (González‐Abraham et al. 2015), indicating these landscapes are too resistant for effective dispersal. According to our predictions, the only cluster of core areas still potentially connected to the Mayan Forest lies in the states of Oaxaca and Chiapas, through a corridor crossing the Isthmus of Tehuantepec (Figure 2k). This region supports important jaguar populations (Lavariega et al. 2020) and could act as a stepping stone linking northern populations from both coasts to those in the south. However, there is a considerable distance between this cluster and the next core areas along both coasts—approximately 160 km to the central Pacific (Figure 2l) and 400 km to the northern Atlantic (Figure 2o)—with the Atlantic side being particularly affected by anthropogenic pressures that further reinforce the isolation predicted by our models (González‐Abraham et al. 2015).

Along the Mexican Pacific coast, three clusters of core areas occur relatively close to one another, yet our model did not identify potential corridors among them (Figure 2l–n). The western edge of the country, particularly in the *Sierra Madre Occidental* and *Sierra Madre del Sur*, retains extensive tracts of natural habitat (González‐Abraham et al. 2015) that could still support jaguar populations or at least function as dispersal routes linking nearby core areas. Nevertheless, previous studies have indicated moderate to low suitability (Alvarenga et al. 2026; Jȩdrzejewski et al. 2018) and low population densities for the species (Charre‐Medellín et al. 2023), with the exception of the coastal mangroves of Nayarit (Luja et al. 2022) and Chamela‐Cuixmala Biosphere Reserve (Núñez‐Pérez 2011). The lack of genetic data further limits deeper conclusions, highlighting the need for future research on population structure in this region. Finally, our models did not identify core areas north of Mexico, and thus no corridors were predicted towards the United States when using the core area approach—although this gap was addressed under the protected area–based corridor analysis (Figures 4 and 5).

### 4.2 Protected Areas Connectivity

Given the accelerating pace of global change affecting natural habitats, especially outside of PAs, we complemented our core area analysis with a protected area–based corridor model to assess potential links among existing PAs across the jaguar's range. This approach proved highly informative, revealing potential connections that the core area approach did not capture. For instance, our results identified the most likely dispersal corridors for the northernmost breeding jaguar population in Sonora, Mexico, extending towards protected areas in New Mexico and Arizona, US (Figures 4 and 5). Detecting these links is important, as dispersing males are occasionally recorded north of the Mexican border, and the region appears to retain suitable habitat for the species (Sanderson et al. 2022). A major obstacle to these movements, and to the potential establishment of a transboundary population, is the US–Mexico border wall, with ongoing construction occurring precisely in the area where our model predicts connectivity is more likely (Chambers et al. 2022). Moving southward, our protected area–based analysis revealed two major shifts in predicted connectivity. The Chiapas‐Mayan Forest corridor disappeared, whereas the Mayan Forest now appears connected across Central America, extending as far south as northern Panama (Figures 4 and 5). These changes likely reflect the distinct configuration of the PAs network, which does not fully overlap with the core areas. While this limits connectivity in southern Mexico, it enhances potential linkages such as those from the Mayan Forest to Honduras, where PAs form a denser mosaic that appears to function as stepping stones connecting both sides. Such results highlight the importance of maintaining and strengthening this network to secure large‐scale functional connectivity across the Mesoamerican Biological Corridor initiative.

In the southern range, another highly relevant and positive change is the emergence of corridors in two key regions. The first connects the Darién Gap to PAs along the Colombia‐Venezuela border, and from there to the Llanos‐Amazon interface (Figures 4 and 5). If confirmed, this connection would represent a remarkable shift in the perspective of jaguar conservation, as it would not only bring significant genetic benefits but also renew hope for restoring connectivity between northern and southern populations. As in the previous model, this network of corridors extends across the Amazon Forest towards the Chaco, Pantanal, Iberá and the Green Corridor and now includes one final, crucial link to the coastal Atlantic Forest of *Serra do Mar*, Brazil (Figure 4i). Such a connection would be extremely valuable for jaguar conservation, and both cases warrant closer investigation to assess their potential effectiveness. On a less positive note, the previously predicted connectivity between the Amazon and the Cerrado‐Caatinga interface was lost in the protected area–based analysis (Figures 4 and 5). As explained earlier, these differences stem from variations in the distribution and concentration of core areas versus PAs. Moreover, the protected area–based analysis assumes the presence of at least one source point (representing a dispersing individual) in each PA, which is unlikely to hold true in all cases. Overall, the differences between the two models highlight regions of greater uncertainty—areas that will likely require increased attention and targeted efforts to maintain or restore functional connectivity. We consider the core area approach to be more realistic than the protected area–based model, as it reflects the actual density and distribution of jaguar occurrence rather than assuming all PAs act as sources of dispersing individuals irrespective of surrounding habitat and population conditions. However, with increasing human pressure, PAs are more likely to persist into the future than unprotected areas, albeit often as increasingly isolated habitat fragments. Thus, using the corridors mapped here to guide long‐term conservation strategies, including the creation of new strategically located PAs, may represent a more pragmatic approach.

### 4.3 Management Priorities and Conservation Implications

This study identified, mapped and ranked the most important core areas, PAs and dispersal corridors for jaguars, providing a framework to inform conservation priorities across the species' range. Given the pronounced ecological heterogeneity within the range, the ranking process accounted for ecoregional distribution. Accordingly, core areas, PAs and corridors intersecting multiple ecoregions were assigned proportionally higher importance, as they contribute to maintaining connectivity across diverse ecological contexts. Therefore, stakeholders and decision‐makers should consider top‐ranked core areas and PAs as those most likely to offer stronger and more stable conditions for the species, while low‐ranked features indicate areas of concern where jaguar populations may be more vulnerable and connectivity weaker. Similarly, the highest‐ranked corridors generally represent broad, well‐connected networks linking multiple strongholds across the landscape.

Among the top‐ranked core areas, our model successfully identified the main regions known to support jaguar populations (Figure 1), aligning with expert knowledge and empirical data (Jȩdrzejewski, Morato, Negrões, et al. 2023; Zeller 2007). Notably, some populations fell just outside the 10 highest‐ranked areas—such as those along Mexico's western edge, the Costa Rica‐Panama border and northern Cerrado in Brazil—although they still appear within the top 20 (full data available in Alvarenga 2026). As discussed previously, these regions harbour important jaguar populations and should not be overlooked in conservation planning. However, many of them experience intense anthropogenic pressures with high rates of habitat conversion, which have reduced core area extent and likely contributed to their lower ranking (e.g., Soterroni et al. 2019).

Among the top‐ranked PAs, most are located within the Amazon biome, reflecting the extensive network of protected areas and Indigenous Lands that has successfully kept much of the region in a near‐pristine state (Pessôa et al. 2023). These Amazonian PAs are pivotal for jaguar conservation, supporting the largest and most stable populations while maintaining strong internal connectivity through several top‐ranked corridors (Figure 5). Outside the Amazon, a number of high‐ranked PAs and corridors—particularly in the Mayan Forest, Central America (Honduras and Nicaragua), the Chaco and the Atlantic Forest—also play critical roles in linking fragmented populations and sustaining regional connectivity. Their inclusion among the top ranks underscores their strategic importance for maintaining the species' functional range.

As expected, the top‐ranked corridors tend to link the highest‐ranked core areas, reflecting regions where jaguar populations are relatively more stable. It is important to note, however, that most jaguar populations outside the central core remain under considerable threat—a pattern that becomes increasingly severe towards the species' range margins (de la Torre et al. 2018). Consequently, high‐ranked corridors in areas such as the Sierra de Tamaulipas (north‐eastern Mexico), the Iberá‐Green Corridor (north‐eastern Argentina) and the coastal Atlantic Forest (Brazil) should be viewed as regionally significant linkages, emphasised here through our ecoregion‐based prioritisation approach. In these areas, the implementation of actions, such as conservation translocations and reintroductions, to connect populations (and restore gene flow) is most urgent.

For low‐ranked core areas, PAs and corridors, targeted interventions, such as habitat restoration, anti‐poaching measures and human‐wildlife conflict mitigation, could improve their contribution to range‐wide connectivity over time. For isolated core areas and PAs, where our models indicate no current connectivity, active restoration of linkages should be considered, particularly where historical habitat loss has severed once‐functional corridors; in such cases, conservation translocations or reintroductions may also be warranted to restore populations and genetic diversity (e.g., Donadio et al. 2022). Many of these isolated areas occur at the species' range margins, where anthropogenic pressure is most intense, and should therefore be treated with urgency as further habitat loss in these regions risks rendering local populations extinct and connectivity irretrievable.

### 4.4 Scope and Limitations

The population connectivity models developed in this study were based on a habitat suitability surface derived from telemetry data of 172 GPS‐collared jaguars distributed across the species' current range. This dataset represents the most comprehensive compilation of jaguar movement information to date, covering individuals from a wide variety of ecosystems and environmental conditions (Alvarenga et al. 2026). As such, it provides a robust foundation for understanding large‐scale connectivity patterns. However, as acknowledged by Alvarenga et al. (2026), the collared individuals were unevenly distributed across the species' range, and future models would certainly benefit from the inclusion of new data, particularly from under‐represented regions such as both Mexican coasts and parts of Central America. As with any modelling effort, our approach is constrained by the spatial and temporal extent of the available data, as well as by assumptions inherent to resistance‐based connectivity models (Unnithan Kumar et al. 2022). These include potential biases in collaring locations and uneven sampling effort across regions, as well as simplifications related to jaguar dispersal behaviour and demographic dynamics. For instance, Alvarenga et al. (2026) used all available telemetry fixes—after appropriate processing—to produce the habitat suitability surface. Because jaguars often move through suboptimal habitats during dispersal (Schoen et al. 2025), we applied a negative exponential transformation to the suitability surface to better represent the non‐linear costs of movement across such landscapes (Keeley et al. 2016). Nonetheless, we recommend that future connectivity models use telemetry data explicitly associated with dispersal behaviour to refine these estimates further. Finally, we manually adjusted the resistance cost of a few landscape features, including cities, roads, elevation and riparian forests. Although resistance layers based solely on expert knowledge can be prone to error and should be avoided when biological data are available (Zeller et al. 2012), minor adjustments such as ours are widely used in the literature (e.g., Brennan et al. 2022; Kaszta et al. 2024; Penjor et al. 2024) and, in our case, were strongly guided by known jaguar responses to these features (e.g., Alegre et al. 2025; Griffith et al. 2021; Morato et al. 2018).

It is important to recognise that models are, by nature, simplifications of reality. The corridors identified here therefore represent potential structural linkages that require field validation and targeted conservation efforts to confirm their functionality—that is, whether they are actively used by jaguars for dispersal and gene flow. Furthermore, given the continental scale of this analysis, the results are best suited for broad spatial prioritisation rather than the design of local conservation interventions.

Site‐specific studies and finer‐scale modelling will be necessary to inform targeted mitigation measures, such as wildlife crossings or habitat management actions, at the local level. Such efforts are already underway in parts of the jaguar's range. In collaboration with Panthera‐Brazil and the World Wide Fund for Nature (WWF‐Bolivia), camera trap surveys have been initiated within selected predicted corridors identified by our models in Brazil and Bolivia. Although these results have not yet been published, both surveys have successfully recorded jaguars within their respective study areas, providing encouraging preliminary support for the predicted corridors. Continued field monitoring, together with future movement and genetic studies, will be essential to determine whether these structural corridors function as dispersal pathways and to refine future iterations of the connectivity models.

## 5 Conclusions

The combination of cumulative resistant kernels and fLCP effectively identified critical areas for jaguar population connectivity across the species' current range. While fLCP corridors provide detailed insights into specific, direct linkages between PAs, cumulative kernels highlight broader core habitat regions for the species. These complementary approaches are especially valuable for diverse landscapes like those across the continent, where tailored conservation strategies are often required. For instance, pinpointing individual corridor lines within current large, continuous forest patches (e.g., the Mayan and Amazonian forests) may be unnecessary, as these areas likely function as effective corridors in their entirety and are therefore better represented as core areas. However, identifying the most probable dispersal pathways in already fragmented and suboptimal habitats of human‐altered landscapes is crucial as these areas are likely to become increasingly fragmented in the future. Prioritising corridors is vital given the limited financial resources. As such, the results of these models should be utilised as complementary tools, leveraging their strengths to address varied ecological challenges.

Encouragingly, our models reveal an extensive central core area in South America that spans parts of 11 countries and encompasses the Llanos‐Amazon‐Pantanal‐Chaco continuum. This region forms the backbone of continental connectivity for the species, supporting the largest, most connected jaguar populations. Coordinated efforts are urgently needed to ensure it remains the demographic and genetic cornerstone of the species. Conversely, many core areas and corridors were identified in regions where connectivity is already severely threatened—such as the Mexican coasts, Central America, southern Paraguay and south‐southeast Brazil. These areas represent key opportunities for targeted restoration, refaunation, and the reinforcement of functional connectivity, particularly where predicted corridors intersect existing protected areas or Indigenous Lands.

At the national level, the prioritisation framework presented here can help guide resource allocation by identifying high‐leverage actions, such as securing narrow linkages between major strongholds or maintaining transboundary corridors that connect populations across borders. Integrating these priorities into broader initiatives, such as the Mesoamerican Biological Corridor and national land‐use planning, will be critical to ensure the long‐term persistence of jaguars under accelerating habitat loss and landscape change.

## Author Contributions

**Guilherme Costa Alvarenga:** conceptualization, investigation, writing – original draft, methodology, validation, visualization, formal analysis, data curation, writing – review and editing. **Caroline C. Sartor:** methodology, writing – review and editing, formal analysis, validation. **Ana Carolina Srbek‐Araujo:** writing – review and editing, validation. **Ana Cristina Mendes‐Oliveira:** writing – review and editing, validation. **Bart Harmsen:** validation, writing – review and editing. **Carlos De Angelo:** validation, writing – review and editing. **Carolina Franco Esteves:** validation, writing – review and editing. **Claudia Bueno de Campos:** validation, writing – review and editing. **Daiana Jeronimo Polli:** validation, writing – review and editing. **Diego F. Passos Viana:** validation, writing – review and editing. **Diogo Maia Gräbin:** validation, writing – review and editing. **Emiliano Donadio:** validation, writing – review and editing. **Emiliano E. Ramalho:** validation, writing – review and editing. **Esteban Payán:** validation, writing – review and editing. **Fernando C. C. Azevedo:** validation, writing – review and editing. **Francisco Palomares:** validation, writing – review and editing. **George V. N. Powell:** validation, writing – review and editing. **Gerardo Ceballos:** validation, writing – review and editing. **Grasiela Porfirio:** validation, writing – review and editing. **Heliot Zarza:** validation, writing – review and editing. **Ivonne Cassaigne:** validation, writing – review and editing. **Juliano A. Bogoni:** validation, writing – review and editing. **Leonardo Sena:** validation, writing – review and editing. **Louise Maranhão:** validation, writing – review and editing. **Marcos Roberto Monteiro de Brito:** validation, writing – review and editing. **Mathias W. Tobler:** validation, writing – review and editing. **Øystein Wiig:** validation, writing – review and editing. **Rafael Antelo:** validation, writing – review and editing. **Rebecca J. Foster:** validation, writing – review and editing. **Ricardo Sampaio:** validation, writing – review and editing. **Robin Naidoo:** validation, writing – review and editing. **Rodrigo Nuñez:** validation, writing – review and editing. **Ronaldo G. Morato:** validation, writing – review and editing. **Sandra Petrone Mendoza:** validation, writing – review and editing. **Valeria Boron:** validation, writing – review and editing. **Wener Hugo Arruda Moreno:** validation, writing – review and editing. **David W. Macdonald:** writing – review and editing, funding acquisition, validation, resources. **Żaneta Kaszta:** conceptualization, methodology, validation, writing – review and editing, project administration, supervision. **Samuel A. Cushman:** methodology, conceptualization, validation, writing – review and editing, supervision, project administration.

## Funding

G.C.A.‘s doctorate was supported by a grant to David Macdonald at WildCRU from the Robertson Foundation. S.A.C. was supported by a grant to David Macdonald from the Dennis Curry Trust and by a NASA project (grant no. 80NSSC25K7244). A.C.S.‐A. thanks the Espírito Santo Research and Innovation Support Foundation (FAPES) for funding the project (FAPES 510/2016), as well as for the Capixaba Researcher Fellowship (FAPES 404/2022).

## Conflicts of Interest

The authors declare no conflicts of interest.

## Supporting information


**Figure S1:** Resistance surface generated using the negative exponential suitability‐to‐resistance transformation of Keeley et al. (2016) with *c* = 0.25. Insets show 10 representative regions illustrating how resistance values vary across landscapes with contrasting habitat conditions. The numbered locations correspond to the resistance values reported in Table S2. Equivalent figures for *c* = 8, 16, and 32 are presented in Figures S2–S4, respectively. Map lines delineate study areas and do not necessarily depict accepted national boundaries.
**Figure S2:** Resistance surface generated using the negative exponential suitability‐to‐resistance transformation of Keeley et al. (2016) with *c* = 8. Insets show 10 representative regions illustrating how resistance values vary across landscapes with contrasting habitat conditions. The numbered locations correspond to the resistance values reported in Table S2. Equivalent figures for *c* = 0.25, 16, and 32 are presented in Figures S1, S3, and S4, respectively. Map lines delineate study areas and do not necessarily depict accepted national boundaries.
**Figure S3:** Resistance surface generated using the negative exponential suitability‐to‐resistance transformation of Keeley et al. (2016) with *c* = 16. Insets show 10 representative regions illustrating how resistance values vary across landscapes with contrasting habitat conditions. The numbered locations correspond to the resistance values reported in Table S2. Equivalent figures for *c* = 0.25, 8, and 32 are presented in Figures S1, S2, and S4, respectively. Map lines delineate study areas and do not necessarily depict accepted national boundaries.
**Figure S4:** Resistance surface generated using the negative exponential suitability‐to‐resistance transformation of Keeley et al. (2016) with *c* = 32. Insets show 10 representative regions illustrating how resistance values vary across landscapes with contrasting habitat conditions. The numbered locations correspond to the resistance values reported in Table S2. Equivalent figures for *c* = 0.25, 8, and 16 are presented in Figures S1, S2, and S3, respectively. Map lines delineate study areas and do not necessarily depict accepted national boundaries.
**Figure S5:** Final resistance surface used in the jaguar ( *Panthera onca* ) population connectivity models. The surface was generated using the negative exponential suitability‐to‐resistance transformation (*c* = 16) and subsequently modified by incorporating the targeted adjustments described in the Methods. Map lines delineate study areas and do not necessarily depict accepted national boundaries.
**Figure S6:** Protected areas, current, and historic jaguar range considered in this study. Map lines delineate study areas and do not necessarily depict accepted national boundaries.
**Figure S7:** Ecoregions (derived from Olson et al. 2001) used in the ranking analysis of core areas, protected areas (PAs), and corridors. Ecoregions 1.1–1.3: Tropical and Subtropical Moist Broadleaf Forests; Ecoregions 2.1–2.4: Tropical and Subtropical Dry Broadleaf Forests; Ecoregions 3: Tropical and Subtropical Coniferous Forests; Ecoregions 7.1–7.3: Tropical and Subtropical Grasslands, Savannas and Shrublands; Ecoregions 8: Temperate Grasslands, Savannas and Shrublands; Ecoregions 9: Flooded Grasslands and Savannas; Ecoregions 10: Montane Grasslands and Shrublands; Ecoregions 13.1, 13.2: Deserts and Xeric Shrublands; Ecoregions 14: Mangroves. Map lines delineate study areas and do not necessarily depict accepted national boundaries.
**Figure S8:** Comparison of alternative cumulative resistant kernel thresholds used to delineate core areas. Core areas derived from the cumulative resistant kernel surface under three threshold scenarios: (a) exclusion of zero values only; (b) exclusion of zero values and values below the 10th percentile within each ecoregion (approach used in this study); and (c) exclusion of zero values and values below the 20th percentile. Excluding the lowest 10% of cumulative kernel values produces only minor changes relative to excluding zero values alone, whereas applying a 20th percentile threshold excludes several areas of recognised importance for jaguar conservation. Map lines delineate study areas and do not necessarily depict accepted national boundaries.
**Table S1:** Reference information of the 172 GPS‐collared jaguars used to predict the habitat suitability surface (Alvarenga et al. 2026), which underpinned the connectivity models.
**Table S2:** Resistance values extracted from 10 representative locations (Figures S1–S4) under eight negative exponential suitability‐to‐resistance transformations (Keeley et al. 2016). The table illustrates how increasing the transformation parameter (*c*) progressively steepens the relationship between habitat suitability and landscape resistance, while maintaining the same resistance range (1–100 cost units).

## Data Availability

The resulting layers of the jaguar range‐wide connectivity model with ranked core areas, PAs and corridors will be made openly available on the Zenodo repository upon manuscript acceptance (Alvarenga 2026): https://doi.org/10.5281/zenodo.18378860.
