# Supplementary material for: Jaguar Range‐Wide Connectivity: Prioritising Core Areas and Corridors Between and Within Protected Areas and Indigenous Lands
Source: Glob Chang Biol. 2026 Sep 4;32(9):e71093. doi: 10.1111/gcb.71093 (PMC13545480; doi:10.1111/gcb.71093)
Supplement: Supplementary file 1 [file GCB-32-e71093-s001.pdf]

Jaguar range-wide connectivity: Prioritizing core areas and corridors between and within Protected Areas and Indigenous Lands

Authors

Guilherme Costa Alvarenga; Caroline C. Sartor; Ana Carolina Srbek-Araujo; Ana Cristina Mendes-Oliveira; Bart Harmsen; Carlos De Angelo; Carolina Franco Esteves; Claudia Bueno de Campos; Daiana Jeronimo Polli; Diego F. Passos Viana; Diogo Maia Gräbin; Emiliano Donadio; Emiliano E. Ramalho; Esteban Payán; Fernando C. C. Azevedo; Francisco Palomares; George V. N. Powell; Gerardo Ceballos; Grasiela Porfirio; Heliot Zarza; Ivonne Cassaigne; Juliano A. Bogoni; Leonardo Sena; Louise Maranhão; Marcos Roberto Monteiro de Brito; Mathias W. Tobler; Øystein Wiig; Rafael Antelo; Rebecca J. Foster; Ricardo Sampaio; Robin Naidoo; Rodrigo Nuñez; Ronaldo G. Morato; Sandra Petrone Mendoza; Valeria Boron; Wener Hugo Arruda Moreno; David W. Macdonald; Żaneta Kaszta; Samuel A. Cushman

Supplementary material

Transformation Parameter "c"

The negative exponential suitability-to-resistance transformation proposed by Keeley et al. (2016) assumes dispersing individuals to move through suboptimal habitats rather than restricting movement exclusively to suitable areas, a pattern documented for jaguars (Schoen et al., 2025), and other carnivores (e.g. Beytell et al., 2024). The transformation is defined as:

$$R = 100–99*((1–\exp(–c*h))/(1–\exp(–c)))$$

where "R" is resistance, "h" is the suitability layer, and "c" determines the strength of the suitability-to-resistance transformation. A value of $c = 0$ results in a linear transformation, whereas increasing "c" values produce progressively steeper negative exponential relationships between habitat suitability and landscape resistance.

To identify an appropriate value for c, we generated resistance surfaces using eight candidate values and compared the resulting spatial patterns (Table S2). Lower values, such as $c = 0.25$ (Figure S1), assigned high resistance values ($R > 70$ cost units) even to extensive areas of continuous natural habitat widely recognised as important for the species, such as the Amazon and the Mayan Forest (Alvarenga et al., 2026; Jędrzejewski et al., 2018). This tendency gradually decreased as "c" increased (Figure S2); however, the resulting resistance surface still assigned relatively high resistance values (e.g. $c = 8$ had $R \approx 15$ cost units) to regions supporting well-established jaguar populations, including the Xingu Indigenous Land (Table S2, location 4) (Alvarenga et al., 2025; Bogoni et al., 2023) and the Iberá National Park (Table S2, location 10) (Donadio et al., 2022). The resistance surface generated with $c = 16$ (Figure S3) produced low and relatively consistent resistance values across Protected Areas (PAs) supporting resident jaguar populations (mean $R = 4.4$ cost units; Table S2, locations 1, 4, 5, 7, 8, and 10), while maintaining a gradual increase in resistance within surrounding buffer zones (mean $R = 15.8$ cost units; Table S2, locations 2, 3, 6, and 9). To explore the effect of a stronger transformation, we tested $c = 32$. The resulting resistance surface assigned uniformly low resistance values (mean $R = 2.3$ across the ten reference locations; Table S2), reducing the contrast among habitat types and effectively treating large portions of the landscape as similarly permeable (Figure S4). Given the limited empirical evidence on habitat selection during dispersal movements (Schoen et al., 2025), the selected transformation ($c = 16$) represents a reasonable compromise between subjective

parameterisation and a reproducible, data-driven approach. The resulting resistance surface reflects the lower risk aversion expected during dispersal while maintaining biologically realistic variation in landscape permeability.

Core areas delineation

To evaluate the influence of the cumulative resistant kernel threshold on core area delineation, we compared three alternative thresholds: (1) exclusion of zero values only, (2) exclusion of zero values followed by the exclusion of values below the 10th percentile within each ecoregion (used throughout the main analysis), and (3) exclusion of zero values, then of values below the 20th percentile. All subsequent processing steps remained identical.

The spatial distribution of core areas was largely unchanged between excluding zero values only and excluding values below the 10th percentile (Fig. S8). Using no percentile threshold increased the total mapped core area from 7.75 to 8.76 million km² (55% to 62% of the current jaguar range) and reduced the number of delineated core areas from 307 to 261, while the largest core area remained dominant, accounting for 98.6% of cumulative kernel values under both scenarios. In contrast, applying a 20th percentile threshold excluded several areas of recognised importance for jaguar conservation, including the Llanos (Jędrzejewski et al., 2023), Pantanal (Alvarenga et al., 2021; Tortato et al., 2021), the Brazilian Savanna (Portugal et al., 2019), and the Gran Chaco (Romero-Muñoz et al., 2019). We therefore retained the 10th percentile threshold as a conservative filter to remove diffuse peripheral areas with very low predicted movement density while preserving the biologically meaningful structure and spatial extent of the core areas.

Figures

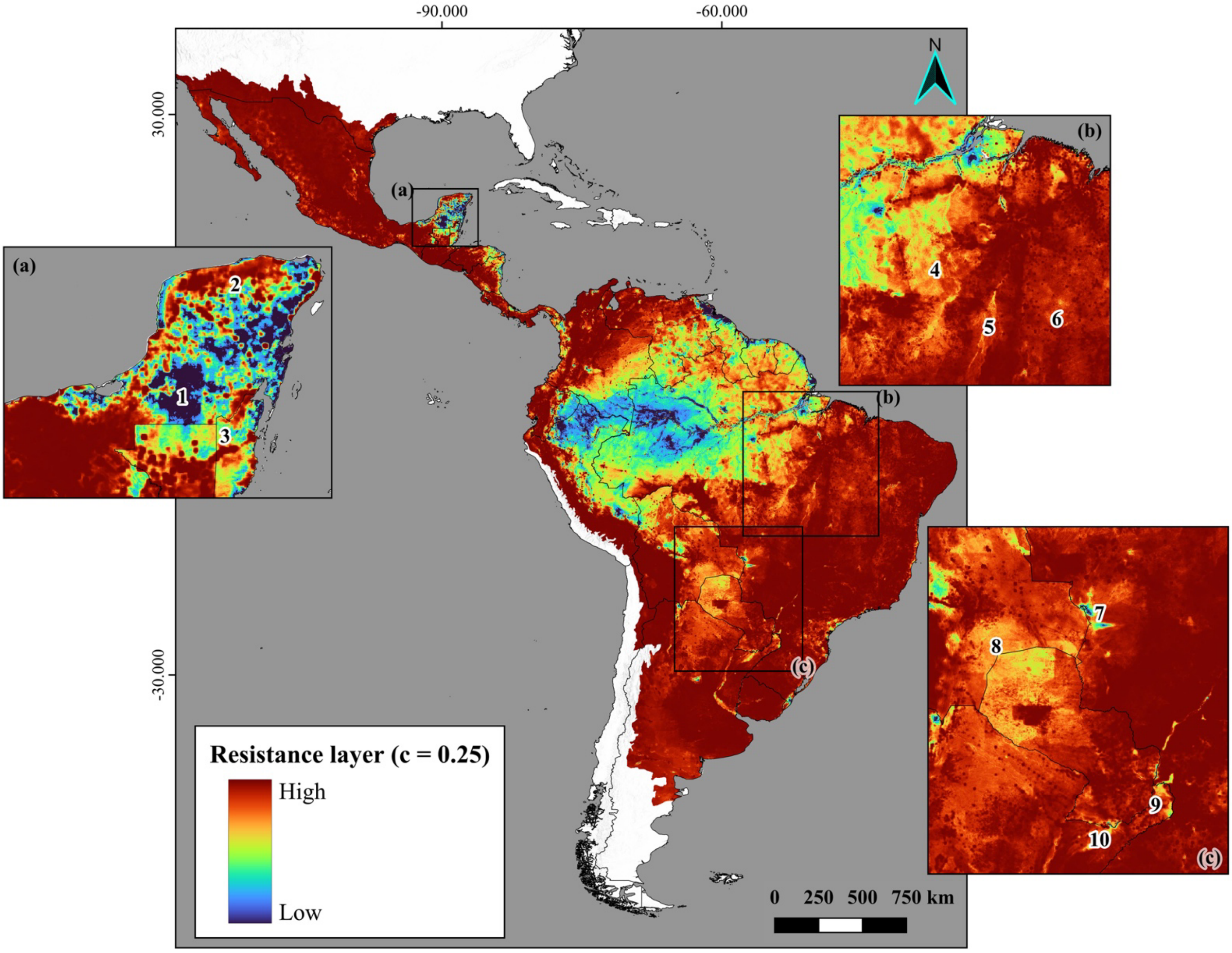

Figure S1. Resistance surface generated using the negative exponential suitability-to-resistance transformation of Keeley et al. (2016) with *c* = 0.25. Insets show ten representative regions illustrating how resistance values vary across landscapes with contrasting habitat conditions. The numbered locations correspond to the resistance values reported in Table S2. Equivalent figures for c = 8, 16, and 32 are presented in Figures S2-S4, respectively. Map lines delineate study areas and do not necessarily depict accepted national boundaries.

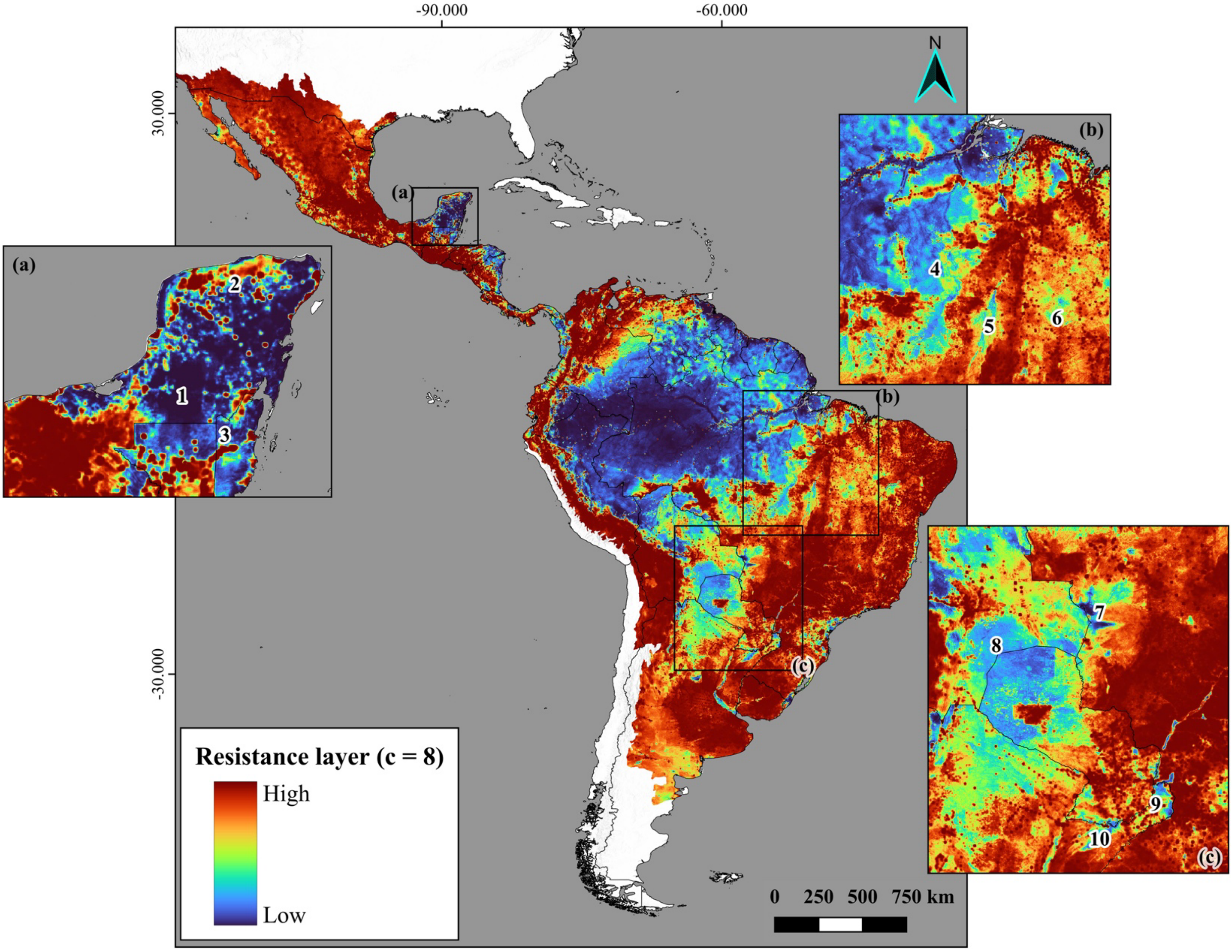

Figure S2. Resistance surface generated using the negative exponential suitability-to-resistance transformation of Keeley et al. (2016) with *c* = 8. Insets show ten representative regions illustrating how resistance values vary across landscapes with contrasting habitat conditions. The numbered locations correspond to the resistance values reported in Table S2. Equivalent figures for c = 0.25, 16, and 32 are presented in Figures S1, S3, and S4, respectively. Map lines delineate study areas and do not necessarily depict accepted national boundaries.

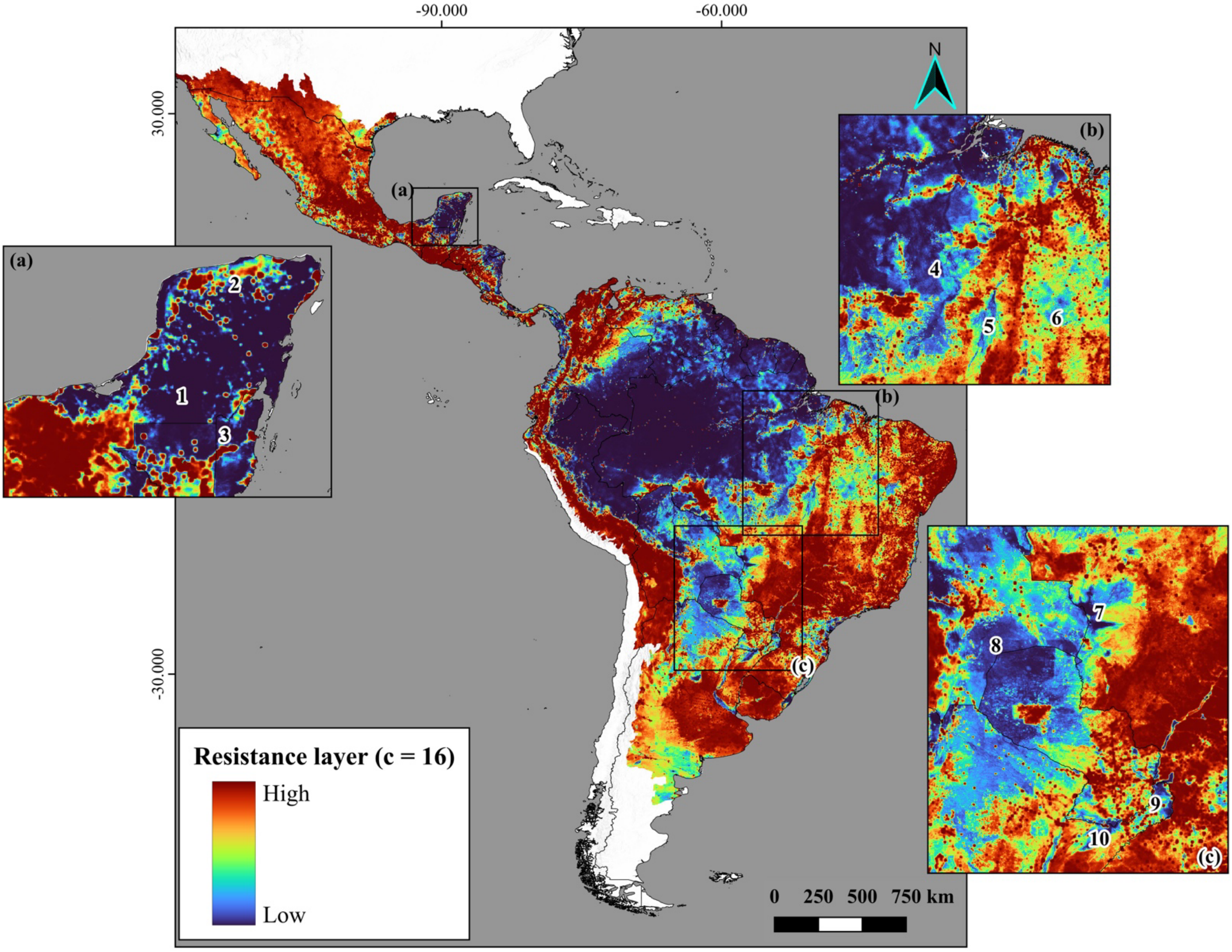

Figure S3. Resistance surface generated using the negative exponential suitability-to-resistance transformation of Keeley et al. (2016) with *c* = 16. Insets show ten representative regions illustrating how resistance values vary across landscapes with contrasting habitat conditions. The numbered locations correspond to the resistance values reported in Table S2. Equivalent figures for c = 0.25, 8, and 32 are presented in Figures S1, S2, and S4, respectively. Map lines delineate study areas and do not necessarily depict accepted national boundaries.

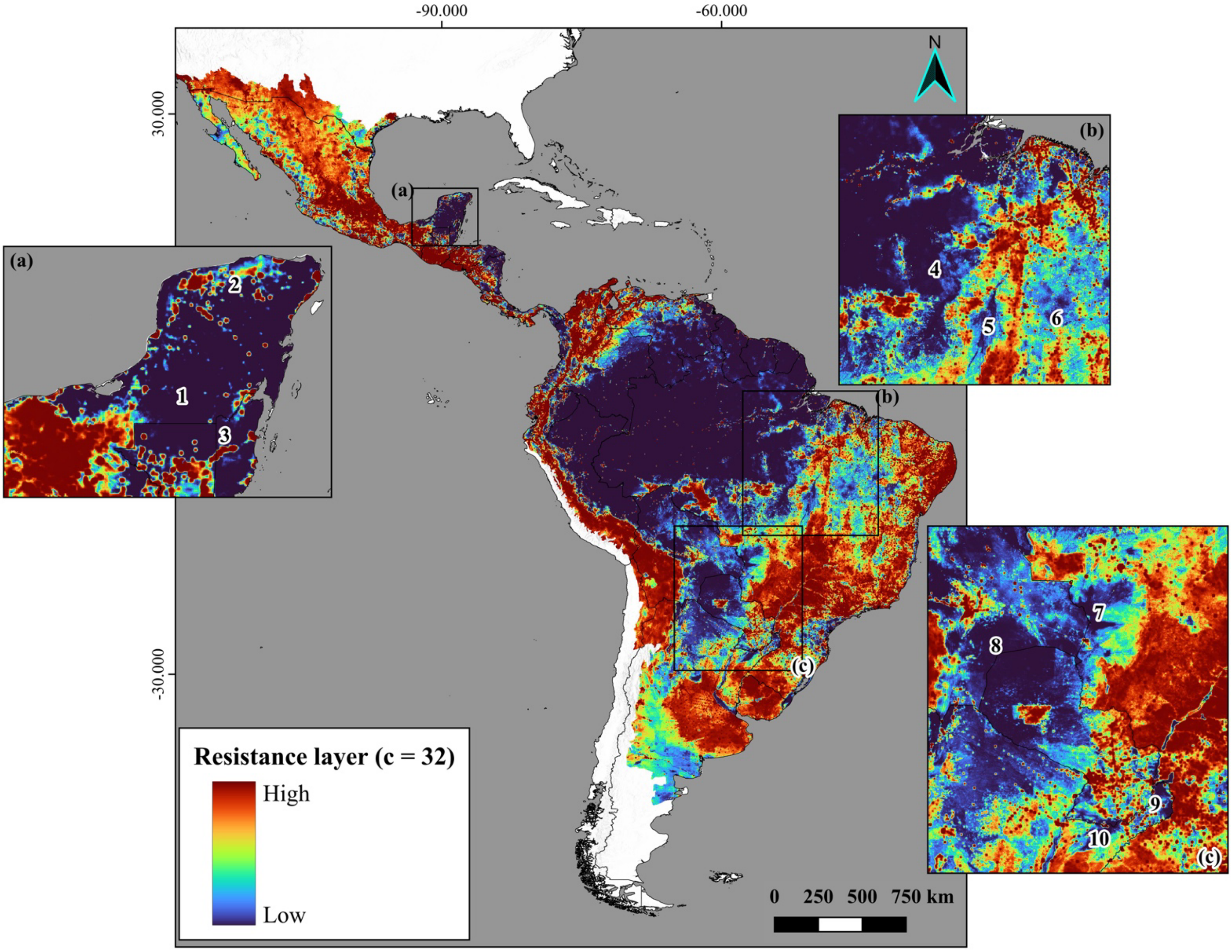

Figure S4. Resistance surface generated using the negative exponential suitability-to-resistance transformation of Keeley et al. (2016) with *c* = 32. Insets show ten representative regions illustrating how resistance values vary across landscapes with contrasting habitat conditions. The numbered locations correspond to the resistance values reported in Table S2. Equivalent figures for c = 0.25, 8, and 16 are presented in Figures S1, S2, and S3, respectively. Map lines delineate study areas and do not necessarily depict accepted national boundaries.

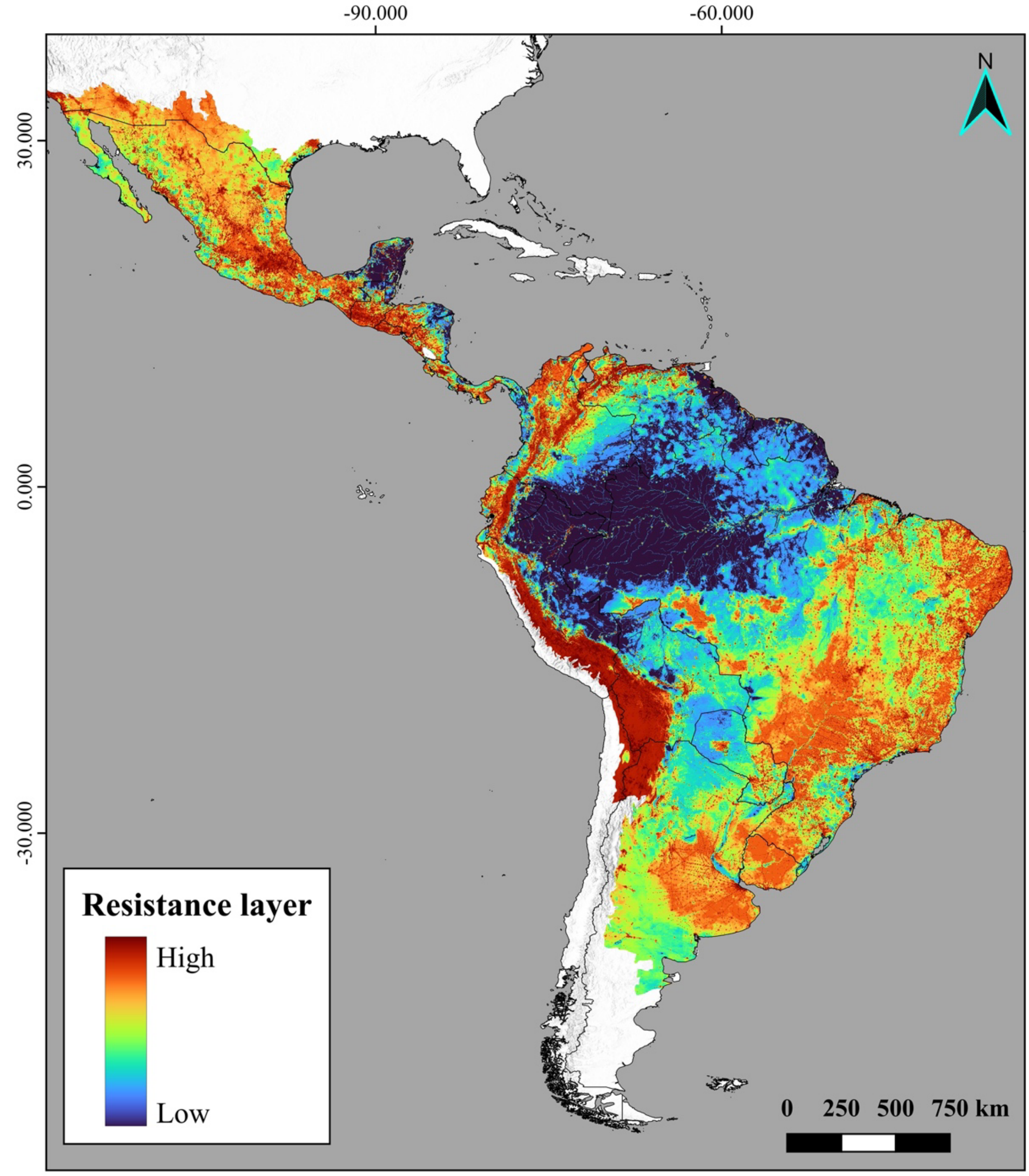

Figure S5. Final resistance surface used in the jaguar (*Panthera onca*) population connectivity models. The surface was generated using the negative exponential suitability-to-resistance transformation (c = 16) and subsequently modified by incorporating the targeted adjustments described in the Methods. Map lines delineate study areas and do not necessarily depict accepted national boundaries.

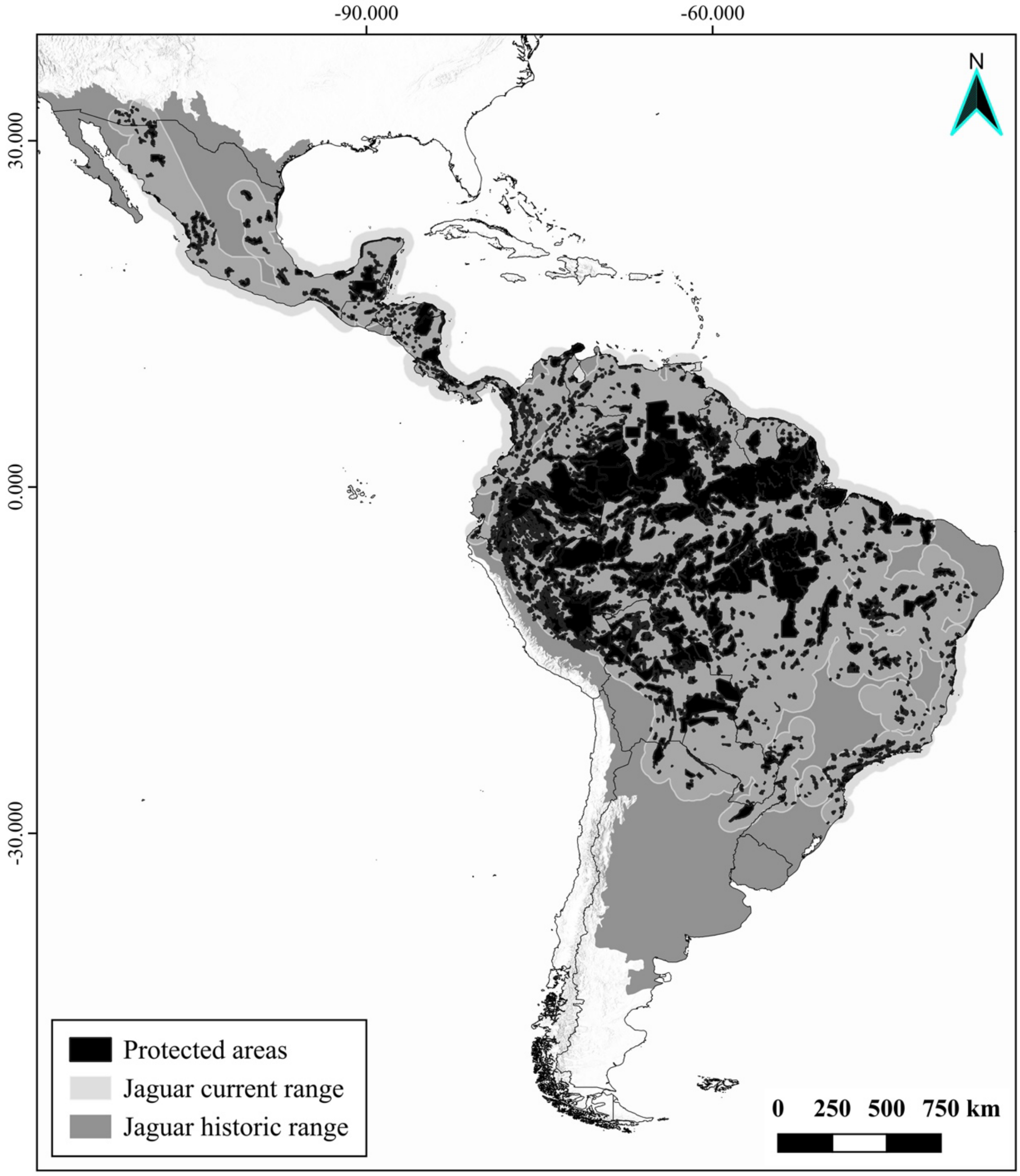

Figure S6. Protected areas, current and historic jaguar range considered in this study. Map lines delineate study areas and do not necessarily depict accepted national boundaries.

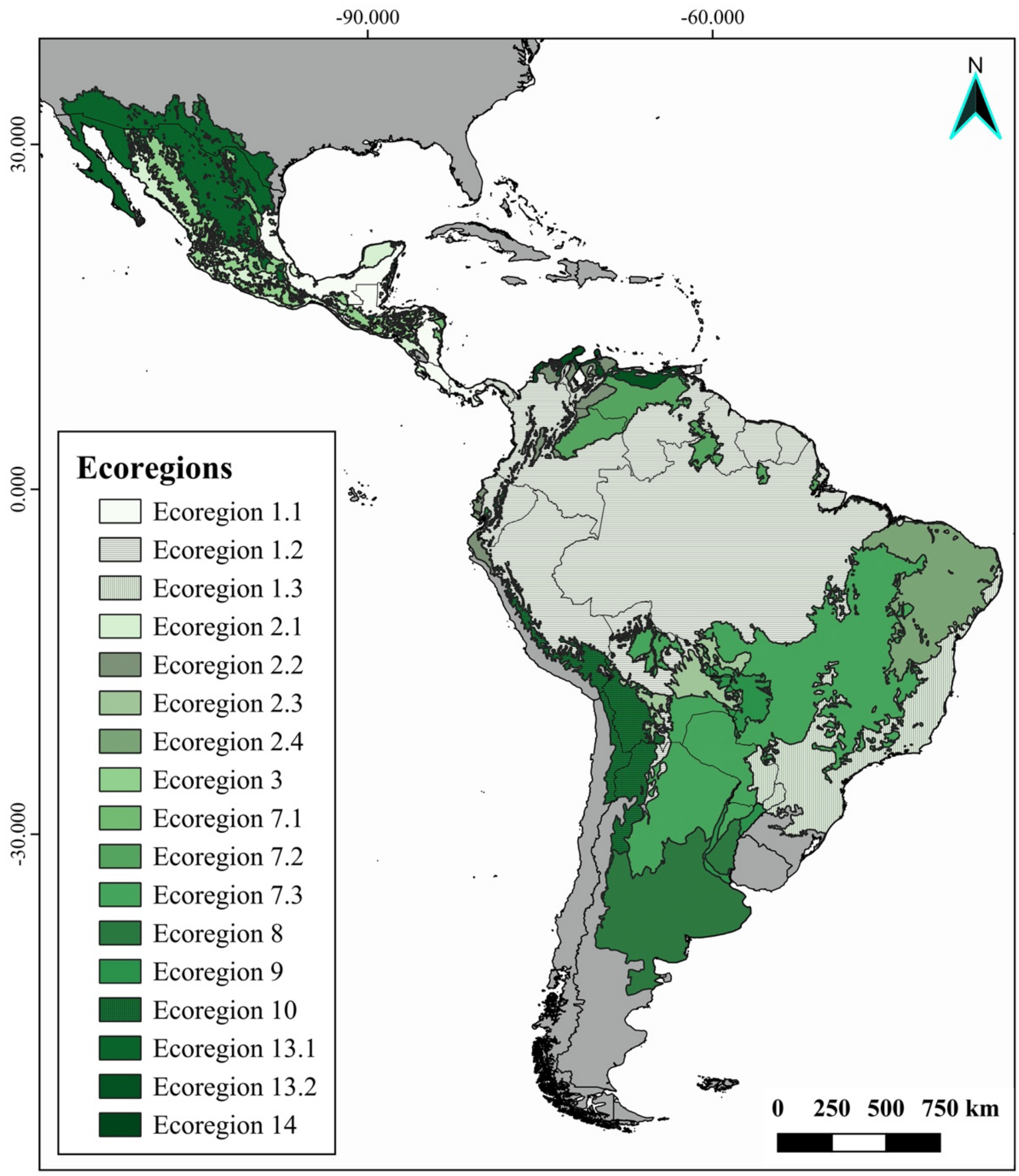

Figure S7. Ecoregions (derived from Olson et al., 2001) used in the ranking analysis of core areas, protected areas (PAs), and corridors. Ecoregions 1.1-1.3: Tropical & Subtropical Moist Broadleaf Forests; ecoregions 2.1-2.4: Tropical & Subtropical Dry Broadleaf Forests; ecoregions 3: Tropical & Subtropical Coniferous Forests; ecoregions 7.1-7.3: Tropical & Subtropical Grasslands, Savannas & Shrublands. ecoregions 8: Temperate Grasslands, Savannas & Shrublands; ecoregions 9: Flooded Grasslands & Savannas; ecoregions 10: Montane Grasslands & Shrublands; ecoregions 13.1, 13.2: Deserts & Xeric Shrublands; ecoregions 14: Mangroves. Map lines delineate study areas and do not necessarily depict accepted national boundaries.

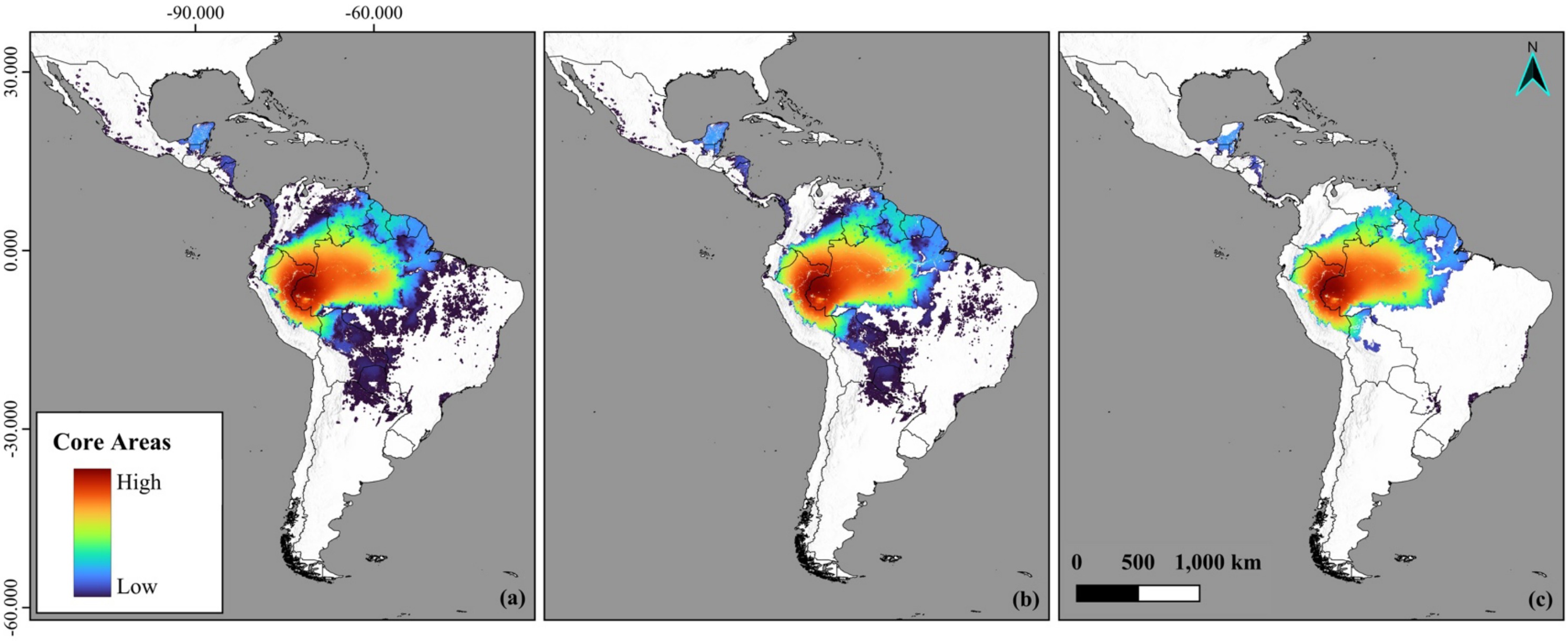

Figure S8. Comparison of alternative cumulative resistant kernel thresholds used to delineate core areas. Core areas derived from the cumulative resistant kernel surface under three threshold scenarios: (a) exclusion of zero values only; (b) exclusion of zero values and values below the 10th percentile within each ecoregion (approach used in this study); and (c) exclusion of zero values and values below the 20th percentile. Excluding the lowest 10% of cumulative kernel values produces only minor changes relative to excluding zero values alone, whereas applying a 20th percentile threshold excludes several areas of recognised importance for jaguar conservation. Map lines delineate study areas and do not necessarily depict accepted national boundaries.

Tables

Table S1. Reference information of the 172 GPS-collared jaguars used to predict the habitat suitability surface (Alvarenga et al., 2026), which underpinned the connectivity models.

Jaguar ID	Sex	Nº Locations	Nº Locations Filter	Tracked Period	Latitude	Longitude	Country
1	F	288	132	06/2010-03/2011	-23.275652	-58.116174	Paraguay
2	F	150	107	07/2010-12/2011	-24.171145	-55.433106	Paraguay
3	M	148	106	06/2009-09/2009	-23.344435	-58.450313	Paraguay
4	F	500	179	07/2011-12/2011	-23.339263	-58.138876	Paraguay
5	F	280	57	07/2009-09/2009	-23.367736	-58.1908264	Paraguay
6	M	983	221	07/2009-12/2009	-23.232139	-58.2325172	Paraguay
7	M	621	207	07/2011-11/2011	-23.25648	-58.077142	Paraguay
8	M	540	196	07/2009-07/2010	-24.040429	-55.486192	Paraguay
9	F	1018	177	06/2014-12/2014	-23.326947	-58.041257	Paraguay
10	F	1668	463	06/2014-05/2015	-23.397239	-58.185852	Paraguay
11	F	913	180	07/2014-07/2015	-23.368049	-58.039784	Paraguay
12	F	2681	261	12/2014-04/2015	-16.9714833	-57.3899431	Brazil
13	M	5040	109	12/2014-08/2015	-16.9159483	-57.4977108	Brazil
14	M	192	64	04/2012-04/2012	-19.8744136	-56.4016528	Brazil
15	M	1323	292	10/2013-01/2014	-20.0301064	-56.3210047	Brazil
16	M	3462	581	04/2011-08/2012	-21.734897	-60.0206985	Paraguay
17	M	537	187	03/2014-07/2014	-17.808023	-53.183055	Brazil
18	M	2314	180	11/2014-04/2015	-16.9279125	-57.4243325	Brazil
19	F	741	111	10/2011-01/2012	-19.9582217	-56.3169619	Brazil
20	M	5590	739	09/2015-06/2016	-8.60977	-42.6801	Brazil
21	F	327	139	08/2011-06/2012	-24.067424	-55.466862	Paraguay
22	M	4709	176	09/2014-05/2015	-16.8864897	-57.4038397	Brazil
23	M	572	38	09/2014-09/2014	-16.8747392	-57.5129536	Brazil
24	M	153	75	12/2011-03/2012	-5.97859	-50.040107	Brazil
25	F	3219	188	10/2012-08/2015	-19.9448036	-56.2427367	Brazil
26	F	5924	333	11/2014-06/2016	10.8596451	-85.6343205	Costa Rica
27	F	560	86	09/2010-11/2013	-17.3260076	-56.7544166	Brazil
28	F	205	65	07/2010-10/2010	-17.828947	-57.40595	Brazil
29	F	67	30	07/2011-09/2011	-17.8398802	-57.3940782	Brazil
30	F	581	125	10/2011-11/2012	-17.3293188	-56.7126546	Brazil
31	F	103	36	10/2013-02/2014	-17.3367322	-56.7099957	Brazil
32	F	240	104	10/2012-04/2013	-17.3793419	-56.7819766	Brazil
33	F	133	68	10/2013-01/2014	-17.3285701	-56.7594096	Brazil
34	F	92	39	02/2003-07/2003	-22.63708499	-52.26445905	Brazil
35	F	17	15	08/2000-11/2001	-22.59397636	-52.25259646	Brazil
36	F	32	28	07/1999-07/2003	-22.5901957	-52.38519834	Brazil
37	F	211	93	07/2002-11/2002	-22.63878999	-52.12664972	Brazil

38	F	18	18	07/2002-08/2002	-22.53828697	-52.25666345	Brazil
39	F	326	187	02/2002-04/2005	-22.75705316	-53.68426924	Brazil
40	F	183	100	05/2004-12/2004	-22.80383586	-53.55541512	Brazil
41	F	4952	90	12/2014-08/2015	-16.8612211	-57.4243539	Brazil
42	M	7668	1047	02/2009-10/2009	-25.637647	-54.299678	Argentina
43	F	1512	556	01/2013-02/2013	29.74605	-109.23039	Mexico
44	F	104	65	01/2012-09/2013	16.835904	-91.042886	Mexico
45	F	39	14	08/2012-08/2012	16.889464	-90.955664	Mexico
46	F	443	250	08/2012-11/2013	16.898824	-91.083115	Mexico
47	M	636	330	01/2012-02/2013	16.579612	-90.999996	Mexico
48	M	72	53	08/2012-12/2012	16.938643	-91.120305	Mexico
49	M	979	280	01/2014-12/2015	21.3790875	-87.6018119	Mexico
50	M	1361	218	11/2014-01/2015	-8.6345197	-42.7049775	Brazil
51	M	727	239	06/2010-12/2010	-20.530443	-58.061521	Paraguay
52	F	615	40	11/2014-12/2014	-16.9132783	-57.4112914	Brazil
53	M	300	148	09/2010-10/2013	-17.265488	-56.8139292	Brazil
54	M	129	22	07/2010-08/2010	-17.8506281	-57.4067729	Brazil
55	M	141	65	06/2011-10/2011	-17.3527833	-56.5766747	Brazil
56	M	109	59	07/2011-10/2011	-17.8231802	-57.5760012	Brazil
57	M	28	8	07/2011-08/2011	-17.8351375	-57.4037579	Brazil
58	M	159	79	01/2003-06/2003	-22.67648014	-52.19555839	Brazil
59	M	434	223	10/2011-12/2013	-17.3239385	-56.7097386	Brazil
60	M	705	187	10/2012-10/2013	-17.3536055	-56.7575736	Brazil
61	M	109	61	06/2013-08/2013	-17.2932905	-56.7014317	Brazil
62	M	156	113	05/2003-10/2003	-22.62201182	-52.20074942	Brazil
63	M	797	409	03/2005-04/2006	-22.74154717	-53.54126196	Brazil
64	M	1694	639	10/2012-12/2012	29.61717	-109.31089	Mexico
65	F	1010	445	03/2014-10/2014	-17.871376	-53.103104	Brazil
66	F	53	41	02/2013-06/2013	-25.6485772	-54.4214814	Brazil
67	F	131	69	03/2014-06/2014	-17.706925	-52.936313	Brazil
68	M	1003	194	11/2011-12/2011	-20.0268575	-56.2552267	Brazil
69	F	3464	317	10/2013-05/2014	-19.9262692	-56.2414756	Brazil
70	M	1094	487	06/2002-06/2003	-22.195088	-60.160038	Paraguay
71	F	921	443	07/2003-07/2004	-19.753344	-59.480243	Paraguay
72	M	722	290	07/2003-07/2004	-19.747937	-59.551036	Paraguay
73	M	620	257	06/2004-09/2004	-20.142131	-59.851225	Paraguay
74	F	1300	185	08/2005-05/2006	-19.971896	-58.217231	Paraguay
75	F	1694	215	08/2005-08/2006	-20.039304	-58.165714	Paraguay
76	F	1610	616	06/2006-07/2007	-20.440784	-60.320772	Paraguay
77	M	1376	576	06/2006-05/2007	-20.51578	-60.33193	Paraguay
78	F	798	292	07/2008-07/2009	-24.522229	-55.34041	Paraguay
79	F	2300	201	04/2015-08/2015	-19.9936717	-56.2941214	Brazil
80	M	479	70	05/2010-07/2010	-25.60912108	-54.49638269	Argentina

81	M	10988	407	10/2013-05/2015	-16.97737	-57.3910972	Brazil
82	M	1335	576	05/2014-12/2015	-17.96005	-53.082933	Brazil
83	M	112	49	01/2012-03/2012	-25.08117226	-53.62542169	Brazil
84	F	4860	227	04/2013-01/2014	-19.95198	-56.3223003	Brazil
85	M	780	400	03/2014-02/2015	-17.690413	-53.107739	Brazil
86	F	1391	276	10/2013-01/2014	-19.9611475	-56.1977572	Brazil
87	F	413	32	05/2012-06/2012	-19.9725481	-56.2818497	Brazil
88	F	1296	116	10/2013-04/2014	-16.8718285	-57.5228518	Brazil
89	M	2432	402	09/2013-10/2015	-15.9061	-48.477581	Brazil
90	F	322	164	08/2012-04/2013	-25.7531786	-54.5845814	Argentina
91	M	88	36	01/2011-01/2012	-16.894062	-57.492675	Brazil
92	F	96	43	01/2011-01/2013	-16.917908	-57.487805	Brazil
93	F	784	144	12/2010-11/2011	-3.050582	-64.852288	Brazil
94	F	3837	244	11/2012-02/2014	-3.054921	-64.845133	Brazil
95	F	542	109	02/2013-07/2013	-2.933995	-64.834189	Brazil
96	M	230	125	01/2013-04/2013	-3.03768	-64.82531	Brazil
97	F	31	3	02/2013-02/2013	-3.037074	-64.843443	Brazil
98	M	61	26	01/2013-09/2013	-3.043342	-64.884696	Brazil
99	M	294	110	10/2013-11/2014	-2.944293	-64.940658	Brazil
100	M	1024	319	03/2014-08/2015	-3.090892	-64.813113	Brazil
101	M	404	207	08/2015-03/2016	-19.710234	-56.357117	Brazil
102	F	151	83	03/2016-05/2016	-19.603761	-56.211355	Brazil
103	F	20	12	06/2014-06/2014	-19.571285	-56.076128	Brazil
104	M	134	59	08/2015-06/2016	-19.578162	-56.151681	Brazil
105	F	2113	251	07/2008-10/2009	-19.51600071	-57.04450005	Brazil
106	M	227	131	09/2009-10/2011	-19.42884891	-56.91394335	Brazil
107	M	287	93	07/2008-12/2008	-19.53291063	-56.78271003	Brazil
108	M	481	197	07/2008-11/2008	-19.46945048	-56.86500405	Brazil
109	F	165	72	08/2008-10/2008	-19.56692044	-56.91692119	Brazil
110	M	166	93	02/2010-05/2010	-19.49668019	-57.12129559	Brazil
111	F	1758	188	07/2008-04/2011	-19.43423998	-56.86302315	Brazil
112	F	202	47	07/2008-10/2008	-19.63714532	-56.9650143	Brazil
113	F	709	140	08/2008-07/2009	-19.47401862	-56.94045639	Brazil
114	F	1645	199	06/2008-07/2009	-19.47993711	-57.08448275	Brazil
115	F	952	247	06/2008-03/2009	-19.53442239	-56.97638914	Brazil
116	M	3340	146	10/2015-04/2016	-16.8496492	-57.5967928	Brazil
117	F	2820	116	10/2015-02/2016	-16.9078125	-57.4846269	Brazil
118	F	451	175	01/2015-01/2016	-3.078417	-64.829936	Brazil
119	M	516	183	01/2015-07/2015	-2.852821	-64.908878	Brazil
120	F	843	137	03/2016-11/2016	-3.042296	-64.856324	Brazil
121	M	643	211	01/2016-10/2016	-3.108568	-64.817603	Brazil
122	M	835	275	02/2017-01/2018	-3.03765	-64.90765	Brazil
123	M	2622	272	03/2018-02/2019	-3.062562	-64.792863	Brazil

124	M	801	146	02/2020-05/2020	-3.021761	-64.964985	Brazil
125	M	4890	436	07/2019-07/2020	-19.14391	-39.99278	Brazil
126	F	4363	771	06/2019-02/2020	-10.76774	-41.69304	Brazil
127	F	1774	343	05/2013-05/2014	18.662806	-89.131262	Mexico
128	F	1221	653	07/2001-03/2009	18.232041	-88.898627	Mexico
129	F	166	125	03/2002-03/2003	18.31096889	-88.95445333	Mexico
130	M	526	72	05/2009-06/2009	18.383318	-89.300211	Mexico
131	M	1315	329	05/2009-07/2009	18.270961	-89.256905	Mexico
132	M	295	207	04/2005-11/2005	18.1338	-89.0362	Mexico
133	F	457	208	04/2005-05/2006	18.176021	-88.888454	Mexico
134	F	307	121	04/2001-01/2002	18.295253	-88.979485	Mexico
135	M	4831	879	01/2021-01/2023	-24.8283769	-61.1156142	Argentina
136	F	1290	190	05/2009-07/2009	18.324955	-89.397159	Mexico
137	M	364	253	06/2002-05/2003	18.215324	-89.558898	Mexico
138	M	1192	244	05/2009-09/2009	18.457128	-89.412146	Mexico
139	F	245	141	04/2008-03/2009	18.231913	-88.898713	Mexico
140	F	286	165	04/2005-08/2007	18.269887	-89.02367	Mexico
141	M	2851	557	10/2012-03/2013	-11.41943858	-69.84469181	Peru
142	M	1618	378	09/2009-12/2009	-11.43448933	-69.77944624	Peru
143	M	189	132	11/2012-02/2013	-11.5411808	-69.7436494	Peru
144	F	903	168	10/2012-12/2012	-11.4049256	-69.85074	Peru
145	F	3848	293	09/2007-02/2008	-13.12314509	-69.59072353	Peru
146	F	3054	323	08/2007-12/2007	-13.12049567	-69.46562018	Peru
147	M	4905	584	07/2012-05/2013	-11.42838766	-69.82209262	Peru
148	F	697	98	09/2007-12/2007	-13.05271558	-69.54118693	Peru
149	M	4199	688	07/2007-02/2008	-12.97864727	-69.58116607	Peru
150	M	2122	172	01/2022-06/2022	-17.925562	-57.476751	Brazil
151	M	13243	1274	08/2017-03/2019	-9.95224	-41.09542	Brazil
152	M	287	80	02/2018-05/2018	5.98766	-71.44483	Colombia
153	F	257	28	02/2018-05/2018	6.0383939	-71.3843953	Colombia
154	M	2555	384	11/2019-06/2020	-3.24246	-47.83422	Brazil
155	F	2361	279	09/2017-07/2018	-19.8004	-42.59652	Brazil
156	M	2174	462	11/2017-11/2018	-19.80304	-42.59469	Brazil
157	F	43	25	07/2021-08/2021	20.9937908	-105.2199662	Mexico
158	F	1257	87	11/2019-03/2020	21.6542017	-105.4480192	Mexico
159	M	296	70	12/2015-03/2016	20.929646	-105.297234	Mexico
160	M	309	44	04/2016-07/2016	21.1989024	-105.2000204	Mexico
161	M	88	41	07/2017-08/2017	21.0400516	-104.9517662	Mexico
162	M	94	37	05/2019-11/2019	20.9081558	-105.3694981	Mexico
163	M	189	131	06/2015-05/2016	19.5015409	-105.0139881	Mexico
164	F	2852	134	10/2021-01/2023	-28.267087	-57.338167	Argentina
165	M	2412	402	01/2022-01/2023	-28.329787	-57.437795	Argentina
166	F	1263	161	04/2021-11/2021	-28.343423	-57.509335	Argentina

167	F	676	100	10/2022-01/2023	-28.337012	-57.441685	Argentina
168	F	5183	450	01/2021-01/2023	-28.243248	-57.302054	Argentina
169	M	1248	318	06/2023-08/2023	-8.691146	-42.521022	Brazil
170	F	3057	426	09/2022-01/2023	-8.647565	-42.67408	Brazil
171	M	362	203	02/2015-02/2016	16.77688791	-88.4860169	Belize
172	M	597	243	02/2015-10/2015	16.7671903	-88.4881106	Belize
TOTAL		231017	37782				

Table S2. Resistance values extracted from ten representative locations (Figures S1-S4) under eight negative exponential suitability-to-resistance transformations (Keeley et al., 2016). The table illustrates how increasing the transformation parameter (c) progressively steepens the relationship between habitat suitability and landscape resistance, while maintaining the same resistance range (1-100 cost units).

**Parameter "c"**	**Reference Figure**	**Resistance Value**									
		**1**	**2**	**3**	**4**	**5**	**6**	**7**	**8**	**9**	**10**
0.25	Figure S1	28.15	88.41	63.56	75.56	77.43	90.37	83.24	76.34	87.68	72.22
0.5	-	25.70	87.14	60.70	73.27	75.26	89.29	81.50	74.10	86.34	69.74
1	-	21.16	84.40	54.89	68.49	70.72	86.96	77.82	69.42	83.46	64.59
2	-	13.74	78.33	43.54	58.56	61.19	81.72	69.87	59.65	77.09	54.08
4	-	5.29	65.43	25.07	40.21	43.22	70.37	53.90	41.45	63.68	35.33
8	Figure S2	1.33	43.75	7.51	17.38	19.88	50.36	30.15	18.38	41.51	13.71
16	Figure S3	1.00	19.47	1.43	3.72	4.61	25.62	9.60	4.06	17.60	2.64
32	Figure S4	1.00	4.45	1.00	1.06	1.13	7.12	1.75	1.09	3.78	1.03

References

Alvarenga, G. C., Chiaverini, L., Cushman, S. A., Dröge, E., Macdonald, D. W., Kantek, D. L. Z., Morato, R. G., Thompson, J. J., Oscar, R. B. L. M., Abade, L., Azevedo, F. C. C. de, Ramalho, E. E., & Kaszta, Ż. (2021). Multi-scale path-level analysis of jaguar habitat use in the Pantanal ecosystem. *Biological Conservation*, *253*, 108900. https://doi.org/10.1016/j.biocon.2020.108900

Alvarenga, G. C., Sartor, C. C., Cushman, S. A., Zimmermann, A., Srbek-Araujo, A. C., Mendes-Oliveira, A. C., Harmsen, B., Angelo, C. De, Esteves, C. F., Campos, C. B. De, Polli, D. J., Viana, D. F. P., Gräbin, D. M., Donadio, E., Ramalho, E. E., Payán, E., Azevedo, F. C. C., Palomares, F., Powell, G. V. N., … Kaszta, Ż. (2026). Range-wide assessment of habitat suitability for jaguars using multiscale species distribution modelling. *Scientific Reports*, *16*, 759. https://doi.org/10.1038/s41598-025-30512-5 2

Alvarenga, G. C., Tobler, M. W., Boron, V., de Carvalho, E. A. R., Morato, R. G., Endo, W., Mendonça, E. N., Sampaio, R., Durigan, C. C., Gonçalves, A. L. S., Ramalho, E. E., Pezzuti, J. C. B., de Paula, M. J., Antunes, A. P., Mena, J. L., Dupont, G., Spironello, W., Pacheco, J., La Rosa-Camino, F., … da Costa, M. O. (2025). Jaguar (*Panthera onca*) density and population size across protected areas and indigenous lands in the Amazon biome, its largest stronghold. *Biological Conservation*, *303*, 111010. https://doi.org/10.1016/j.biocon.2025.111010

Beytell, P., Hanssen, L., Aschenborn, O., & Naidoo, R. (2024). Long-Distance, Transfrontier Carnivore Dispersals in Southern Africa. *Ecology and Evolution*, *14*(11), 1–7. https://doi.org/10.1002/ece3.70574

Bogoni, J. A., Boron, V., Peres, C. A., Coelho, M., Morato, R. G., & Oliveira-da-Costa, M. (2023). Impending anthropogenic threats and protected area prioritization for jaguars in the Brazilian Amazon. *Communications Biology*, 1–11. https://doi.org/10.1038/s42003-023-04490-1

Donadio, E., Zamboni, T., & Martino., S. Di. (2022). Bringing jaguars and their prey base back to the Iberá wetlands, Argentina. In et al. Gaywood M. (Ed.), *Conservation Translocations* (pp. 443–448). Cambridge University Press.

Jędrzejewski, W., Boron, V., Garrido, E. P., Hoogesteijn, R., Abarca, M., Romero, A. P., Viloria, Á., Lampo, M., Marquina, F., & Velásquez, G. (2023). Jaguars (*Panthera onca*) in the Llanos of Colombia and Venezuela: Estimating Distribution and Population Size by Combining Different Modeling Approaches. In G. P. Mandujano, S., Naranjo, E.J., Andrade Ponce (Ed.), *Neotropical Mammals*. Springer, Cham. https://doi.org/https://doi.org/10.1007/978-3-031-39566-6_9

Jędrzejewski, W., Robinson, H. S., Abarca, M., Zeller, K. A., Velasquez, G., Paemelaere, E. A. D., Goldberg, J. F., Payan, E., Hoogesteijn, R., Boede, E. O., Schmidt, K., Lampo, M., Viloria, Á. L., Carreño, R., Robinson, N., Lukacs, P. M., Nowak, J. J., Salom-Pérez, R., Castañeda, F., … Quigley, H. (2018). Estimating large carnivore populations at global scale based on spatial predictions of density and distribution - Application to the jaguar (*Panthera onca*). *PLoS ONE*, *13*(3), 1–25. https://doi.org/10.1371/journal.pone.0194719

Keeley, A. T. H., Beier, P., & Gagnon, J. W. (2016). Estimating landscape resistance from habitat suitability: effects of data source and nonlinearities. *Landscape Ecology*, *31*(9), 2151–2162. https://doi.org/10.1007/s10980-016-0387-5

Olson, D. M., Dinerstein, E., Wikramanayake, E. D., Burgess, N. D., Powell, G. V. N., Underwood, E. C., D'Amico, J. A., Itoua, I., Strand, H. E., Morrison, J. C., Loucks, C. J., Allnutt, T. F., Ricketts, T. H., Kura, Y., Lamoreux, J. F., Wettengel, W. W., Hedao, P., & Kassem, K. R. (2001). Terrestrial ecoregions of the world: A new

map of life on Earth. *BioScience*, *51*(11), 933–938. https://doi.org/10.1641/0006-3568(2001)051[0933:TEOTWA]2.0.CO;2

Portugal, M. P., Morato, R. G., Ferraz, K. M. P. M. D. B., Rodrigues, F. H. G., & Jacobi, C. M. (2019). Priority areas for jaguar *Panthera onca* conservation in the Cerrado. *Oryx*, *54*(6), 854–865. https://doi.org/10.1017/S0030605318000972

Romero-Muñoz, A., Torres, R., Noss, A. J., Giordano, A. J., Quiroga, V., Thompson, J. J., Baumann, M., Altrichter, M., McBride, R., Velilla, M., Arispe, R., & Kuemmerle, T. (2019). Habitat loss and overhunting synergistically drive the extirpation of jaguars from the Gran Chaco. *Diversity and Distributions*, *25*(2), 176–190. https://doi.org/10.1111/ddi.12843

Schoen, J. M., DeFries, R., & Cushman, S. (2025). Open-source, environmentally dynamic machine learning models demonstrate behavior-dependent utilization of mixed-use landscapes by jaguars (*Panthera onca*). *Biological Conservation*, *302*, 110978. https://doi.org/10.1016/j.biocon.2025.110978

Tortato, F. R., Hoogesteijn, R., Devlin, A. L., Quigley, H. B., Bolzan, F., Izzo, T. J., Ferraz, K. M. P. M. B., & Peres, C. A. (2021). Reconciling biome-wide conservation of an apex carnivore with land-use economics in the increasingly threatened Pantanal wetlands. *Scientific Reports*, *11*(1), 1–8. https://doi.org/10.1038/s41598-021-02142-0
